# Can a single ammonia and water molecule enhance the formation of methanimine under tropospheric conditions?: kinetics of ^•^CH_2_NH_2_ + O_2_ (+NH_3_/H_2_O)

**DOI:** 10.3389/fchem.2023.1243235

**Published:** 2023-09-21

**Authors:** Manas Ranjan Dash, Mohamad Akbar Ali

**Affiliations:** ^1^ Department of Chemistry, School of Physical Sciences, DIT University, Dehradun, Uttarakhand, India; ^2^ Department of Chemistry, College of Art and Science, Khalifa University of Science and Technology, Abu Dhabi, United Arab Emirates; ^3^ Advanced Materials Chemistry Center (AMCC), Khalifa University of Science and Technology, Abu Dhabi, United Arab Emirates

**Keywords:** aminomethyl radical, O_2_ radical, methanimine, *ab initio*/DFT, RRKM/ME, H_2_O and NH_3_, HCN, catalysis

## Abstract

The aminomethyl (•CH_2_NH_2_) radical is generated from the photo-oxidation of methylamine in the troposphere and is an important precursor for new particle formation. The effect of ammonia and water on the gas-phase formation of methanimine (CH_2_NH) from the ^•^CH_2_NH_2_ + O_2_ reaction is not known. Therefore, in this study, the potential energy surfaces for ^•^CH_2_NH_2_ + O_2_ (+NH_3_/H_2_O) were constructed using *ab initio*//DFT, i.e., *coupled*-*cluster theory* (CCSD(T))//hybrid-density functional theory, i.e., M06-2X with the 6-311++G (3df, 3pd) basis set. The Rice−Ramsperger−Kassel−Marcus (RRKM)/master equation (ME) simulation with Eckart’s asymmetric tunneling was used to calculate the rate coefficients and branching fractions relevant to the troposphere. The results show 40% formation of CH_2_NH at the low-pressure (<1 bar) and 100% formation of CH_2_NH_2_OO^•^ at the high-pressure limit (HPL) condition. When an ammonia molecule is introduced into the reaction, there is a slight increase in the formation of CH_2_NH; however, when a water molecule is introduced into the reaction, the increase in the formation of CH_2_NH was from 40% to ∼80%. The calculated rate coefficient for ^•^CH_2_NH_2_ + O_2_ (+NH_3_) [1.9 × 10^−23^ cm^3^ molecule^−1^ s^−1^] and for CH_2_NH_2_ + O_2_ (+H_2_O) [3.3 × 10^-17^ cm^3^ molecule^-1^ s^-1^] is at least twelve and six order magnitudes smaller than those for free ^•^CH_2_NH_2_ + O_2_ (2 × 10^−11^ cm^3^ molecule^−1^ s^−1^ at 298 K) reactions, respectively. Our result is consistent with that of previous experimental and theoretical analysis and in good agreement with its isoelectronic analogous reaction. The work also provides a clear understanding of the formation of tropospheric carcinogenic compounds, i.e., hydrogen cyanide (HCN).

## 1 Introduction

Methylamine is a simple organic nitrogen compound that is released into the atmosphere from a range of sources, for example, food industries, animal husbandry, marine sources, and biomass burning (Schade and Crutzen, 1995; Ge et al., 2011a; Ge et al., 2011b; Zhang et al., 2012; Almeida et al., 2013). Methylamine forms a particulate salt when reacting with acids such as H_2_SO_4_, HNO, and CH_3_COOH; therefore, it plays a vital role in enhancing atmospheric cloud nucleation (Murphy et al., 2007; Lee and Wexler, 2013). The reaction of methylamine with various tropospheric oxidants such as O_3_, OH, and NO_3_ radicals leads to the formation of semi-volatile and non-volatile chemical species, consequently leading to the formation of secondary organic aerosols (Schade and Crutzen, 1995; Murphy et al., 2007; Ge et al., 2011a; Nielsen et al., 2012; Qiu and Zhang, 2013). Methylamine is also expected to be present in the interstellar medium (ISM), which leads to the formation of amino acids (Altwegg et al., 2016; Elsila et al., 2009; Gonza´lez et al., 2022). Although glycine (HO_2_CCH_2_NH_2_) has not yet been identified in the ISM medium, it is detected in different comets (Elsila et al., 2009; Altwegg et al., 2016). Methylamines are also possible atmospheric precursors of hydrogen cyanide and nitrous oxide (N_2_O) (Nielsen et al., 2012). N_2_O is a greenhouse gas and the potential source of stratospheric NOx production. To know the significance of methylamine reactions in the two drastically different environments, several researchers have investigated their atmospheric significance and sinks in both the gas phase and solid phase (Schade and Crutzen, 1995; Ge et al., 2011a; Ge et al., 2011b; Almeida et al., 2013).

**FIGURE 1:**
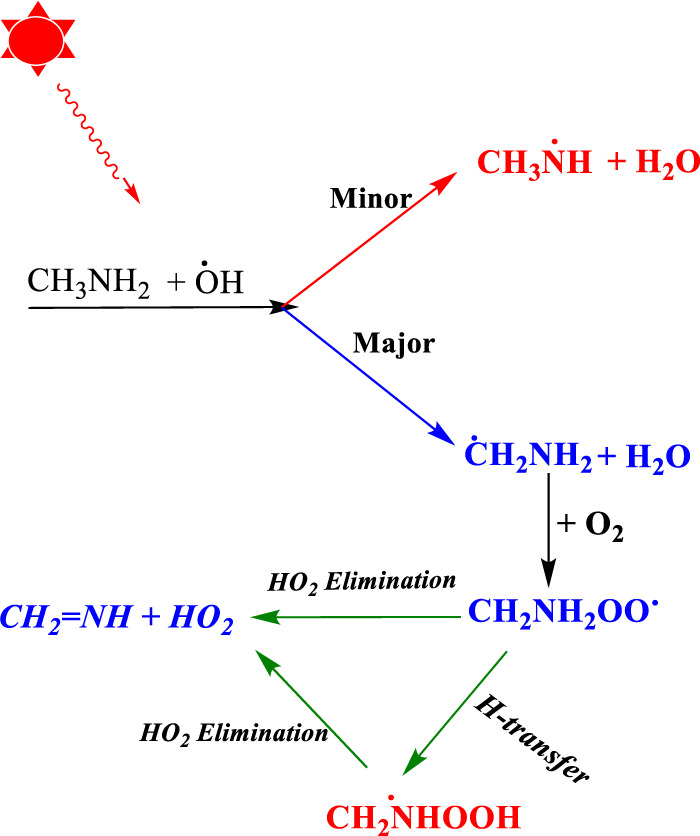
Photo-oxidation mechanism of methylamine (Gonza´lez et al., 2022; Onel et al., 2014; Rissanen et al., 2014; Ashraful and Silva, 2020).

Once CH_3_NH_2_ is released into the Earth’s atmosphere, it reacts with the OH radical via the H-abstraction reaction, leading to the formation of a carbon-centered aminomethyl (^•^CH_2_NH_2_) radical, which is observed as a major product, and nitrogen-centered methyl amino radical (CH_3_NH^•^), which is observed as a minor product (Figure 1) (Gonza´lez et al., 2022; Onel et al., 2014; Rissanen et al., 2014; Ashraful and Silva, 2020).

As suggested in the previous studies (Gonza´lez et al., 2022; Onel et al., 2014; Rissanen et al., 2014; Ashraful and Silva, 2020), ^•^CH_2_NH_2_ predominantly reacts with molecular oxygen (O_2_), which can lead to the formation of methanimine (CH_2_NH) and the hydroperoxy radical (HO_2_) as major reaction products via a hydrogen atom transfer (HAT) mechanism (Gonza´lez et al., 2022; Onel et al., 2014; Rissanen et al., 2014; Ashraful and Silva, 2020). The chemical kinetics studies on the ^•^CH_2_NH_2_ + O_2_ reaction system have been investigated by various researcher groups (Masaki et al., 1995; Jansen et al., 1999; Rissanen et al., 2014; Ashraful and Silva, 2020; Glarborg et al., 2020). Jansen et al. (1999) used pulse radiolysis and UV-absorption detection to analyze the chemical kinetics of the ^•^CH_2_NH_2_ + O_2_ reaction at 298 K and 1 atm of SF_6_ as a bath gas. Masaki et al. (1995) investigated the kinetics of the same reaction by employing the photoionization mass spectrometry technique at 298 K and a few torr pressure of N_2_. Rissanen et al. (2014) used the laser flash photolysis technique in combination with photoionization mass spectrometry to determine the rate coefficients of the ^•^CH_2_NH_2_ + O_2_ reaction. They observed negative temperature-dependent rate coefficients from 267 K to 363 K, independent of the pressure between 0.5 Torr and 2.5 Torr (Rissanen et al., 2014). The reported rate coefficients fall in the range of (2–8) × 10^–11^ cm^3^ molecule^−1^ s^-1^ (Rissanen et al., 2014; Mallick et al., 2018; Ashraful and Silva, 2020; Kumar et al., 2020). Rissanen et al. (2014) also performed quantum chemical calculations coupled with ME simulation to predict the product branching fractions. Their modeling results reproduce the experimentally observed negative temperature dependence and validated the production of CH_2_NH under low-pressure conditions. Recently, a chemical kinetic model for the oxidation of methylamine has been characterized by Glarborg et al. (2020) and validated against the results obtained from shock tube experiments. In their work, the potential energy surface of several reactions was studied theoretically starting from the isomerization of CH_3_NH and the reactions of CNH*x* (*x* = 3–5) molecules with O_2_ using the quantum chemistry composite method. In this study, the rate coefficients for ^•^CH_2_NH_2_ + O_2_ were re-investigated at high-level quantum chemical calculations with similar statistical rate theories to validate the finding for the role of ammonia and water and molecules in the same reaction. To the best of our knowledge, the branching ratios and temperature- and pressure-dependent rate coefficients have not been available until now.

Concerning the gas-phase reactivity of ^•^CH_2_NH_2_ toward O_2_ in the role of ammonia and water, several studies in the past few years have proposed the role of different species such as H_2_O, NH_3_, formic acid, and CO_2_ on important atmospheric reactions (Vöhringer-Martinez et al., 2007; Iuga et al., 2010; Buszek et al., 2012; Iuga et al., 2011; Thomsen et al., 2012; Jonas et al., 2013; Zhang et al., 2013; Zhang et al., 2014; Jara-Toro et al., 2017; Ali et al., 2018; Inaba, 2018; Mallick et al., 2018; Ali, 2019; Ali et al., 2019; Ali, 2020; Kumar et al., 2020; Wu et al., 2020; Zhang et al., 2020; Ali et al., 2021; Ali and Balaganesh, 2022; Ali et al., 2022; Dash and Ali, 2022). It is well-known that ammonia (NH_3_) is highly alkaline and is one of the most common chemicals used in the agriculture sector, and as a fertilizer, it is the major source in the atmosphere. Ammonia is mainly produced industrially and exists naturally as a product of the decomposition of organic matter. It is also used as a refrigerant gas and in the production of plastics, textiles, dyes, explosives, and other chemicals. The emissions of NH_3_ into the Earth’s atmosphere have been increasing over the last few decades. The change in NH_3_ concentration has essential implications for air quality and the ecosystem. To ascertain the significant influence of NH_3_ on many atmospheric reactions, several researchers have investigated the role of NH_3_ on many important atmospheric reactions (Jonas et al., 2013; Mallick et al., 2018; Ali, 2019; Kumar et al., 2020; Zhang et al., 2020; Ali et al., 2021). To this end, it is essential to clearly understand the reaction between ^•^CH_2_NH_2_ and O_2_ in the presence of NH_3_, given its similar catalytic efficiency to water.

Water vapor is an environmentally significant constituent of the Earth’s atmosphere. Numerous investigations have been carried out to determine the catalytic role of a single H_2_O molecule in many atmospheric and combustion reaction systems (Vöhringer-Martinez et al., 2007; Iuga et al., 2010; Buszek et al., 2012; Iuga et al., 2011; Thomsen et al., 2012; Zhang et al., 2013; Zhang et al., 2014; Jara-Toro et al., 2017; Ali et al., 2018; Inaba, 2018; Ali et al., 2019; Ali, 2020; Wu et al., 2020; Ali and Balaganesh, 2022; Ali et al., 2022; Dash and Ali, 2022). These studies reveal that water-catalyzed reactions are energetically more favorable than other catalyzed reactions due to the formation of many hydrogen-bonded intermediates and transition states. However, water does not enhance the reaction’s rate coefficients under tropospheric conditions due to its high concentration and lower entropic contribution compared to a free reaction (Ali et al., 2019; Ali, 2020; Ali and Balaganesh, 2022; Dash and Ali, 2022).

In this paper, we have investigated the rate coefficients for the effect of NH_3_ and H_2_O molecules on the important atmospheric and combustion prototype reactions, i.e., ^•^CH_2_NH_2_ + O_2_, for the first time. Using the RRKM/ME simulation, the temperature- and pressure-dependent rate coefficients were calculated between 200 K and 400 K and pressure ranges of 0.0001–1000 atm. The role of enthalpy and entropy contributions on hydrogen-bonded species on the effect of ammonia and water on the ^•^CH_2_NH_2_ + O_2_ reaction has been discussed to understand the chemical kinetic behavior of these complexes. In these situations, we have been inspired to model a gas-phase ternary reaction system, ^•^CH_2_NH_2_ ···O_2_···X (X = NH_3_, H_2_O), where H_2_O and NH_3_ can act as catalysts (*vide Infra*). To assess the accuracy of the data provided in this work, we have compared the energies and re-calculated rate coefficients and compared them with the available literature data for ^•^CH_2_NH_2_ + O_2_ and its isoelectronic similar reaction, i.e., ^•^CH_2_OH + O_2_ (Dash and Ali, 2022). We hope that this study will strengthen the chemical kinetic database for global modeling and provide a thorough understanding for further study on analogous reaction systems.

## 2 Theoretical and computational methodology

### 2.1 Quantum chemical calculations

All the electronic structure calculations were carried out with the Gaussian 09 suite of programs (Frisch, 2013). The stationary points on potential energy surfaces (PESs) for ^•^CH_2_NH_2_ + O_2_, ^•^CH_2_NH_2_ + O_2_ (+NH_3_), and ^•^CH_2_NH_2_ + O_2_ (+H_2_O) reactions were computed using the hybrid-density functional method, i.e*.*, M06-2X (Zhao and Truhlar, 2008) with the Pople 6-311++G (3df, 3pd) basis set (Frisch et al., 1984) and tabulated in Supplementary Table S1. The M06-2X is a frequently used preeminent functional to investigate the non-covalent interactions of transition states, intermediates, and post-intermediates for investigating chemical systems that encounter hydrogen bonding. To add corrections from the van der Waals interaction on M06-2X (Zhao and Truhlar, 2008), the Grimme empirical dispersion “GD3” was used (Grimme et al., 2010). Normal modes of the vibrational frequency for each optimized species were carried out to obtain the zero-point energy (ZPE) and to calculate the rotational–vibrational partition functions. The transition state (TS) shows a single imaginary frequency, whereas reactants, intermediates, and products all show positive vibrational frequencies (see Supplementary Table S2). Intrinsic reaction coordinate (IRC) calculations (Fukui, 1981) were performed to confirm the identity of intermediates and post-intermediates for each TS. The IRC calculation was performed in both directions with the maxpoints=50 and the step size set to 3. The internal degrees of freedom of all species involved in the reaction were treated as harmonic oscillators and rigid rotor approximations, as suggested in previous studies for similar reaction systems (Ali et al., 2018; Dash and Ali, 2022). To improve the accuracy of energy, the single-point energy calculations were carried out at CCSD(T)/6-311++G (3df, 3dp)//M06-2X/6-311++G (3df, 3dp)+GD3 (Frisch et al., 1984; Raghavachari et al., 1989; Zhao and Truhlar, 2008). The result provides values that are accurate enough up to ∼1 kcal/mol, as validated in our previous studies (Ali et al., 2019; Ali, 2020; Ali and Balaganesh, 2022; Dash and Ali, 2022). To check the qualitative contribution of the single-reference wave function, we have carried out the T1 diagnostic calculation at CCSD(T)/6-311++G (3df, 3pd). The calculated T1 diagnostic was found to be ≤ 0.03, which is an acceptable range for a single reference wave function. To understand the spin contamination for each species, the spin expectation value <S^2^> was calculated and found to be in the range of ∼0.75–0.77, which indicates that spin contamination was negligible.

### 2.2 State-of-the-art kinetics calculations

All the kinetics calculations were carried out using a software tool in the MultiWell suite of the program (Barker, 2009; Barker, 2011; Barker, 2023). The “*me*” codes in MultiWell programs calculate the unimolecular rate coefficients *k(E*) based on the RRKM/master equation as follows (Forst, 2003):

$$k\left(E\right)=\left[\frac{m^{\neq }}{m}\frac{\sigma_{ext}}{\sigma_{ext}^{\neq }}\right]\frac{g_{e}^{\neq }}{g_{e}}\frac{1}{h}\frac{G^{\neq }\left(E-E_{0,0}\right)}{\rho \left(E\right)}.$$ (1)

To avoid repetition from the previous studies, the details of each term of the equation are given in Supplementary Material S1. To calculate temperature- and pressure-dependent rate coefficients and branching fractions, N_2_ bath gases were used with an approximate value of the energy transfer process <*ΔE* > _down_ = 200 × (T/300)^0.85^ cm^−1^ (Goldsmith et al., 2012). The Lennard–Jones parameters for collider gases (N_2_) εk_B_, σ(N_2_) = 3.74 Å, and ε/k_B_(N_2_) = 82 K were obtained from Hippler et al. (1983). The Lennard–Jones parameters of NH_2_CH_2_O_2_ and NH_2_CH_3_O_2_ were approximated based on Rissanen et al. (2014). The double arrays used in *me* simulations consisted of 1500 array elements with 10 cm^-1^ energy grains using a quasi-continuum regime, which is evaluated up to 85,000 cm^-1^. At each pressure and temperature value, ME simulations were carried out using the chemical activation energy distribution, which is appropriate for association reactions. The RRKM/ME simulations consisted of 10^5^ stochastic trials, each with a simulated time duration corresponding to an average of 100 collisions.

The pressure-dependent total rate coefficients $k^{bimol}\left(T,M\right)$ for ^•^CH_2_NH_2_ + O_2_ were calculated using (Ali, 2020; Dash and Ali, 2022)

$$k^{bimol}\left(T,M\right)={\Gamma \;K}_{eq}\times k_{\infty }^{uni}\left(1-f_{{CH}_{2}{NH}_{2}+O_{2}}\right),$$ (2)

where ( Γ ) is the quantum mechanical tunneling correction to the microcanonical rate coefficients *k(E)*. Γ was implemented in the MultiWell master equation code, which is based on the 1-D Eckart asymmetric barrier. The *k(E)* calculated using the modified sums of states of the transition state reflect the tunneling effects. Tunneling was used to initialize the chemical activation distribution if both the “CHEMACT” and “TUN” keywords were selected. The $f_{{CH}_{2}{NH}_{2}+O_{2}}$ is the branching fraction (*f*) of the reaction going back to the reactants, and $k_{\infty }^{uni}$ is a high-pressure limit rate coefficient. The *fall-off* behavior of rate coefficients from (pressure = 1000 bar, P→∞) toward the low-pressure limit (*p* = 0.0001 bar, P→0) was considered.

For the barrierless reactions, i.e., ^•^CH_2_NH_2_ + O_2_→ CH_2_NH_2_OO, ^•^CH_2_NH_2_ … H_2_O + O_2_→ CH_2_NH_2_OO … H_2_O, ^•^CH_2_NH_2_ … NH_3_+O_2_→^•^CH_2_NH_2_OO … NH_3_, the inverse Laplace transform (ILT) method was used. Since the rate coefficients for association reactions are usually weak and dependent on temperature, the activation energy for the recombination reaction was assumed to be equal to 0. As suggested in many similar reactions (Rissanen et al., 2014), this approach is good, and Arrhenius’s activation energy can be equal to the reaction critical energy (E_0_). MultiWell input for ILT calls for only two parameters E_0_ and A-factor. In this work, we use statistical rate theories, which do not account for non-statistical effects, such as slow intramolecular vibrational energy redistribution (IVR), as suggested by Ali et al. (2023).

The equilibrium constant (K_*eq*_) for the formation of ^•^CH_2_NH_2_ + O_2_→ CH_2_NH_2_OO^•^, ^•^CH_2_NH_2_ … H_2_O→CH_2_NH_2_OO … H_2_O, ^•^CH_2_NH_2_ … NH_3_→^•^CH_2_NH_2_OO … NH_3_,^•^CH_2_NH_2_ … H_2_O + O_2_→CH_2_NH_2_OO … H_2_O and ^•^CH_2_NH_2_ … NH_3_+O_2_→^•^CH_2_NH_2_OO … NH_3_ was calculated using the “THERMO” code as given: (Barker, 2009; Barker, 2011; Barker, 2023)

$$K_{eq}=\frac{Q_{\mathrm{INT}}}{Q_{R}}\exp\left(-\frac{E_{\mathrm{INT}}-E_{R}}{k_{B}T}\right).$$ (3)

The equilibrium constants ( $K_{eq}$ ) for the formation of two-body and three-body complexes calculated by Eq. 3 are tabulated in Supplementary Tables S3, S4. The $Q_{\mathrm{INT}}$ and $Q_{R}$ are total partition functions of the intermediates and reactants, respectively; $E_{\mathrm{INT}}-E_{R}$ is the zero-point corrected energy difference between intermediates and reactants. The calculated rate coefficients in the high-pressure limit ( $k_{\infty }$ ) were fitted to the modified Arrhenius expression $k_{\infty }\left(T\right)=A\times T^{n}\times \text{ex}p\left(\frac{-E_{a}}{RT}\right)$ in the temperature range of 200 K–400 K.

## 3 Results and discussion

### 3.1 Geometries and Energies

#### 3.1.1 Reaction channels for ^•^CH_2_NH_2_ + O_2_

**FIGURE 2:**
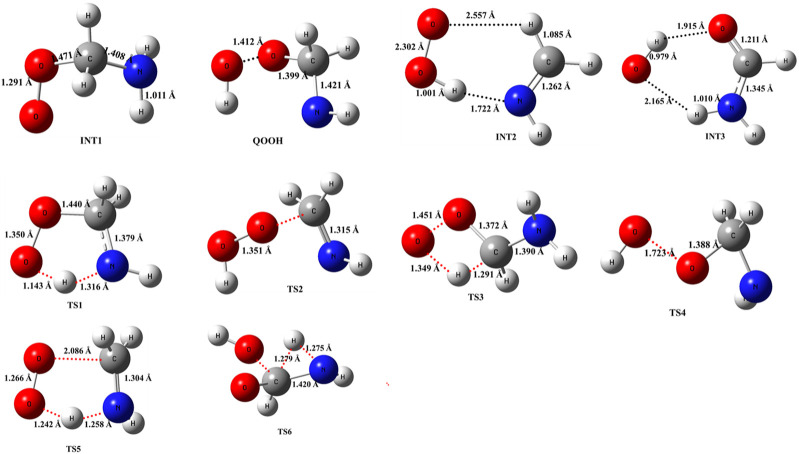
Structural and geometrical changes during the ^•^CH_2_NH_2_ + O_2_ reaction calculated using M06-2X/6-311++G (3df, 3pd).

**FIGURE 3:**
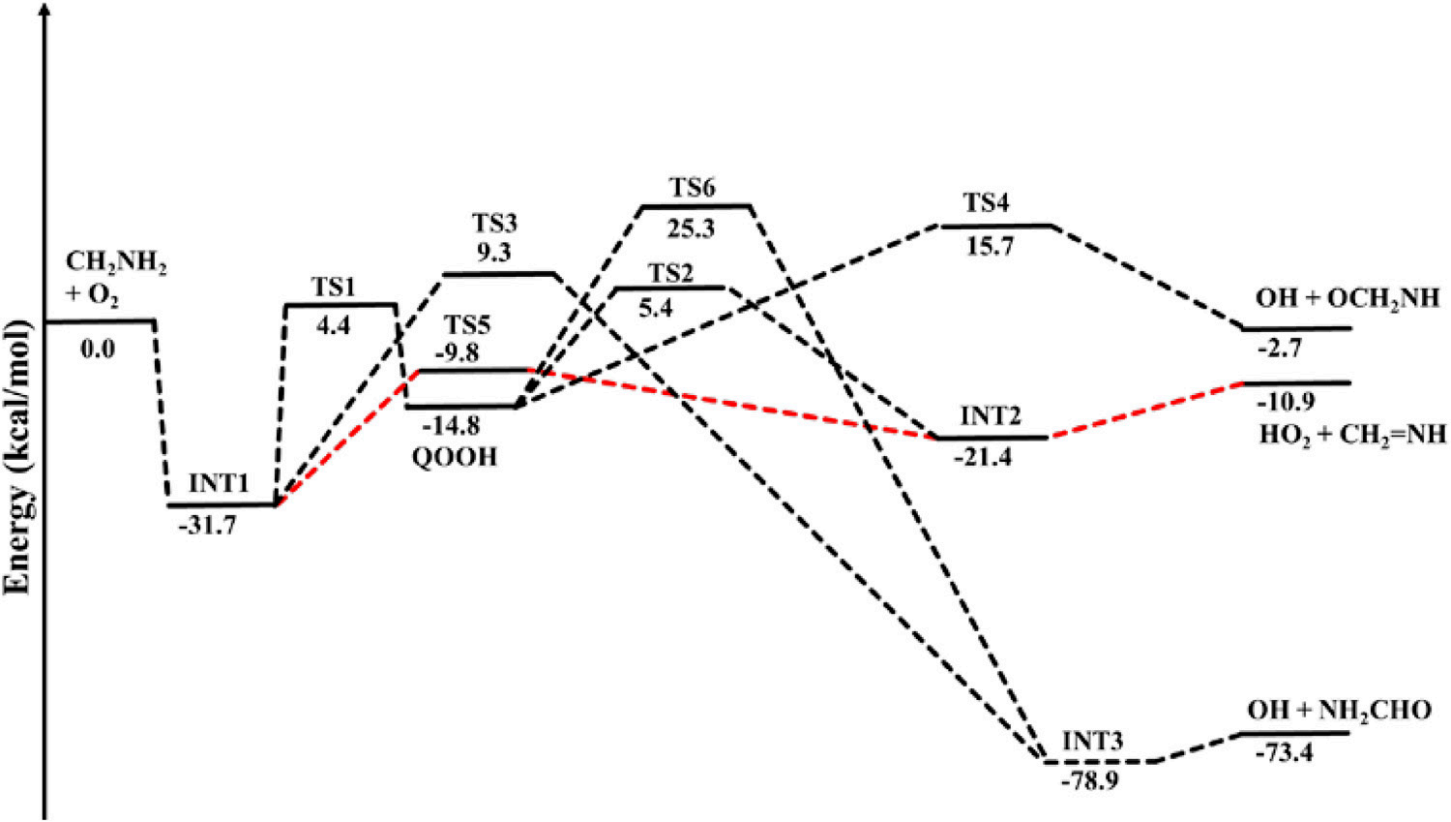
Potential energy surface for the ^•^CH_2_NH_2_ + O_2_ reaction obtained using CCSD(T)/6-311++G (3df, 3pd)//M06-2X/6-311++G (3df, 3pd). The energies shown in the figure include the zero-point energy.

The optimized structures of intermediates and transition states are shown in Figure 2. The zero-point corrected PES for the ^•^CH_2_NH_2_ + O_2_ reaction is depicted in Figure 3, and enthalpies values are given in Table 1. In the current reaction system, the O_2_ molecule attacks the radical carbon atom, which leads to the formation of the intermediate ^•^OO-CH_2_NH_2_ (INT1). Several conformational isomers of INT1 were observed, and for simplicity, we have considered the lowest energy conformer in our calculation. The calculated stabilization energy for INT1 is −31.7 kcal mol^-1^, which is in very good agreement with the reported values by Rissanen et al. (2014) and Zhang et al. (2020) This value is also in very good agreement with its isoelectronic reactions, i.e., the O_2_ + ^•^CH_2_OH value (−31.9 kcal/mol) (Dash and Ali, 2022). The unpaired electron in INT1 resides at the terminal O-atom, which can be decomposed differently. The lowest energy channel is an isomerization process where the terminal O-atom attacks the H-atoms of the NH_2_ group via five- and six-membered cyclic transition states (TS5 and TS1), leading to hydrogen-bonded five- and six-membered cyclic complexes, i.e., INT2 and QOOH, respectively. The calculated barrier heights for TS5 and TS1 are 21.9 and 36.1 kcal mol^-1^, respectively, with respect to INT1, indicating that the isomerization reaction going through TS5, leading to the formation of INT2, is energetically more favorable than that going through TS1 to QOOH. Supplementary Figure S1 provides an IRC scan that confirms the connectivity of TS5 with INT2 and CH_2_NH + HO_2_ at the M06-2X/6-311++G (3df, 3pd). It is noted that the influence of the formation of hydrogen-bonded cyclic complexes may change the energetics and kinetics of the reaction system. In INT2, two strong hydrogen bonds are formed between the H-atom of the HO_2_ and the N-atom of the CH_2_NH (1.72 Å) and O-atom of the HO_2_ radical and the H-atom of the CH_2_NH (2.55 Å), which leads to the formation of a stable six-membered ring planar cyclic structure. In QOOH, a five-membered ring cyclic structure with one hydrogen bond is formed between the terminal O and H-atoms (2.47 Å). Therefore, INT2 is energetically 6.6 kcal mol^-1^ more stable than QOOH. QOOH can further dissociate via TS2 and TS4 to form HO_2_ + CH_2_NH and OH + OCH_2_NH, respectively. Because the barrier height for the formation of QOOH is very high, the formation of CH_2_NH and OCH_2_NH via QOOH may be negligible under tropospheric conditions. The terminal O-atom of INT1 can also attack the H-atom of a nearby C-atom, leading to the formation of a hydrogen-bonded six-membered cyclic intermediate, INT3, via a four-membered ring transition state (TS3) and subsequently dissociating to form OH + NH_2_CHO. In INT3, two strong hydrogen bonds are formed between the H-atom of the OH radical and the O-atom of the NH_2_CHO (1.91 Å) and the O-atom of the OH radical and the H-atom of the NH_2_CHO (2.16 Å). INT3 is energetically the most stable structure in the PES, with a stabilization energy of −78.9 kcal mol^-1^ from the reactants. The barrier height of this reaction channel is 41 kcal mol^-1^, which is 20 kcal mol^-1^ higher than that of the TS5 and may not contribute to the overall reaction kinetics (*vide infra*).

**TABLE 1:** Comparison of enthalpies (in kcal mol^-1^) of each species for the ^•^CH_2_NH_2_ + O_2_ reaction with those found in previous studies and its isoelectronic analogs.

^•^CH_2_NH_2_ + O_2_ →	*This Work*	*Previous works*. Rissanen et al. (2014); Glarborg et al. (2020)	^•^CH_2_OH + O_2_ →	*Previous Work* (Dash and Ali, 2022)	∆*S*_*r,298K*_
H_2_NCH_2_OO (INT1)	−31.7	−33.3^a^,-32.5^b^	HOCH_2_OO (Int-1)	−31.9	−37.3
H···NHCH_2_OO (TS1)	4.4	2.5^a^	H···OCH_2_OO (TS-1)	−7.4	−39.6
HO_2_···CH_2_NH (TS2)	5.4	2.7^a^	HO_2_···CH_2_O (TS-2)	2.8	−36.2
H···CHNH_2_OO (TS3)	9.3	6.2^a^	HO···HCOOH (TS-3)	8.6	−38.0
HO···OCH_2_NH (TS4)	15.7	12.4^a^	HO···OCH_2_O (TS-4)	24.0	−35.3
H···NHCH_2_···OO (TS5)	−9.8	−10.2^a^,-12.0^b^	H···O_2_··CH_2_O (TS-5)	−18.5	−38.9
H···NHCHO···OH (TS6)	25.3	21.8^a^	-	-	−35.9
HO···OCH_2_NH (QOOH)	−14.8	−16.1^a^	HO···OCH_2_O	−14.2	−37.6
OOH···NHCH_2_ (INT2)	−21.4	−22.8^a^,22.0^b^	OOH···OCH_2_ (Int-2)	−25.6	−29.2
HO···NH_2_CHO (INT3)	−78.9	−77.2^a^	-	-	−29.3
CH_2_NH + HO_2_	−10.9	−12.2^a^,-10.6^b^	CH_2_O+ HO_2_	−17.9	1.8
NH_2_CHO + OH	−73.4	−73.1^a^			−2.0
OCH_2_NH + OH	−2.7	−2.9^a^	OCH_2_O+ OH	−15.0	−5.5

^a^ Rissanen et al. (2014).

^b^ Glarborg et al. (2020).

The other reaction channel, such as the conversion from QOOH to INT3 via H-atom shift (C to N) through a three-membered transition state, TS6 (41 kcal mol^−1^), is expected to have a negligible impact on the total rate coefficient due to its high energy barriers.

The enthalpies of reaction (ΔH_rxn_ (0 K) for ^•^CH_2_NH_2_ + O_2_ → CH_2_NH + ^•^HO_2_ (−10.9 kcal mol^-1^) are in very good agreement with those found in the most accurate active thermochemical database (ATcT) (−11.42 kcal mol^-1^) (Ruscic et al., 2004; Ruscic and Bross, 2020) and in good agreement with the theoretically calculated value in Rissanen et al. (2014) (−12.2 kcal mol^-1^). The computed PES for the ^•^CH_2_NH_2_ + O_2_ reaction is also consistent with its isoelectronic analogous reaction system, i.e., ^•^CH_2_OH + O_2_ reported by Dash and Ali (2022) using CCSD(T)//ωB97XD/6-311++G (3df, 3pd) level of theory. The enthalpy values obtained in their calculations are also given in Table 1. The reaction energies for CH_3_NH^•^ + O_2_ → CH_2_NH + HO_2_ (−17.3 kcal/mol) are also calculated and found to be in very good agreement with that in the ATcT (−17.9 kcal/mol) (Ruscic et al., 2004; Ruscic and Bross, 2020). The energies obtained for most of the structures of the ^•^CH_2_OH + O_2_ system (Ali, 2020) along the reaction paths are very close to those of the current system, indicating the reliability of the data presented here. However, the barrier heights for the isomerization pathways Int-1→TS-1 (−7.4 kcal/mol)→QOOH and Int-1 →TS-5 (−18.5 kcal/mol) Int-2 in the CH_2_OH + O_2_ system (Dash and Ali, 2022) are quite low and more stable compared to those in the same pathways in the current system with respect to the reactant’s energy (Table 1). Moreover, the barrier height for the reaction proceeding from HO···OCH_2_O to OCH_2_O + OH (Dash and Ali, 2022) is 24 kcal/mol (TS-4), and the stabilization energy for the products is −15 kcal/mol, whereas in the current system, the corresponding energies are 8.3 and 12.3 kcal/mol less stable than the former ones, respectively. These differences in barrier energies can affect the overall rate coefficients between the two systems.

#### 3.1.2 Role of the ammonia molecule on ^•^CH_2_NH_2_ + O_2_

**FIGURE 4:**
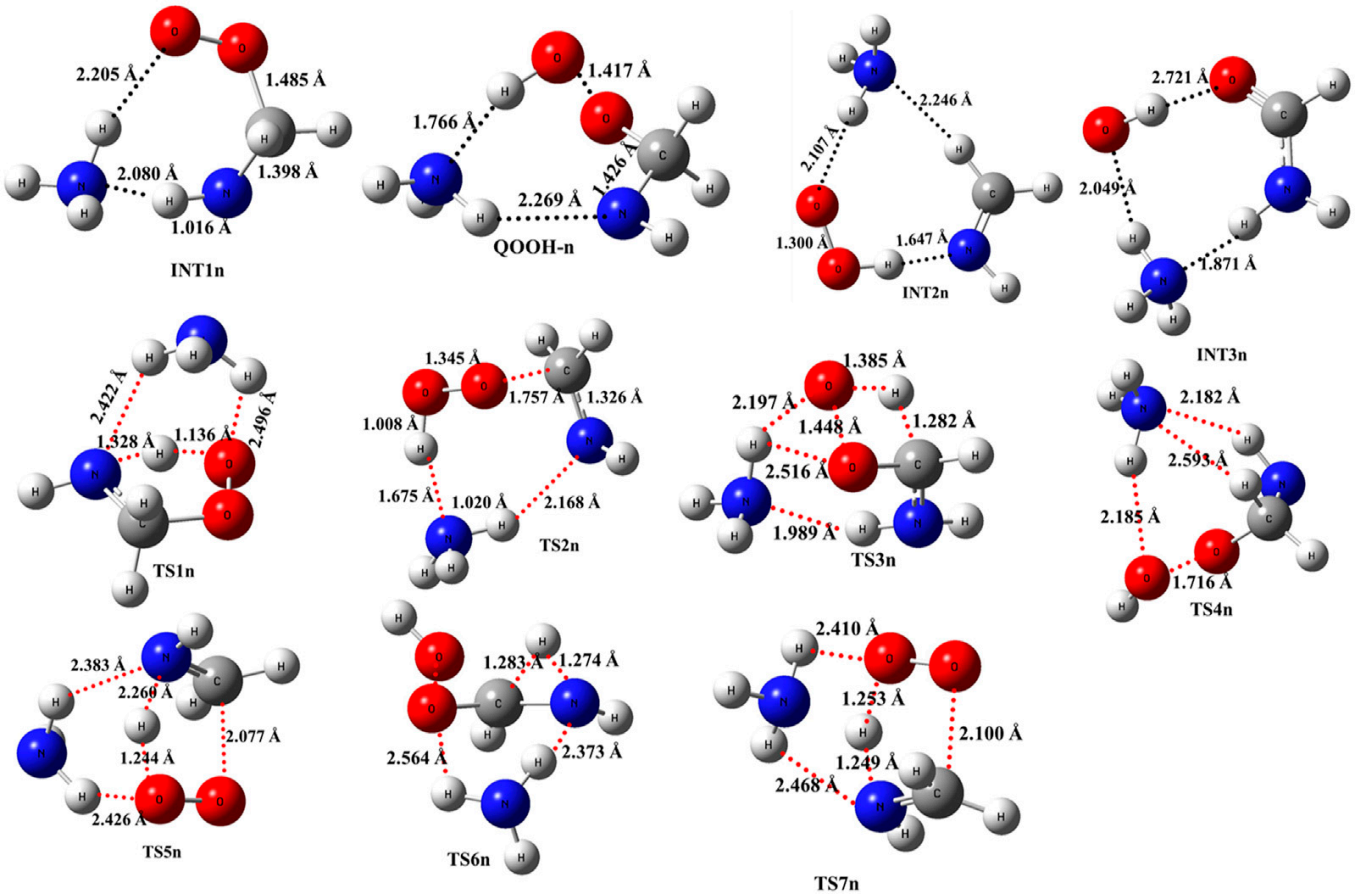
Structural and geometrical changes for the ammonia-assisted ^•^CH_2_NH_2_ + O_2_ reaction calculated using M06-2X/6–311++G (3df, 3pd).

**FIGURE 5:**
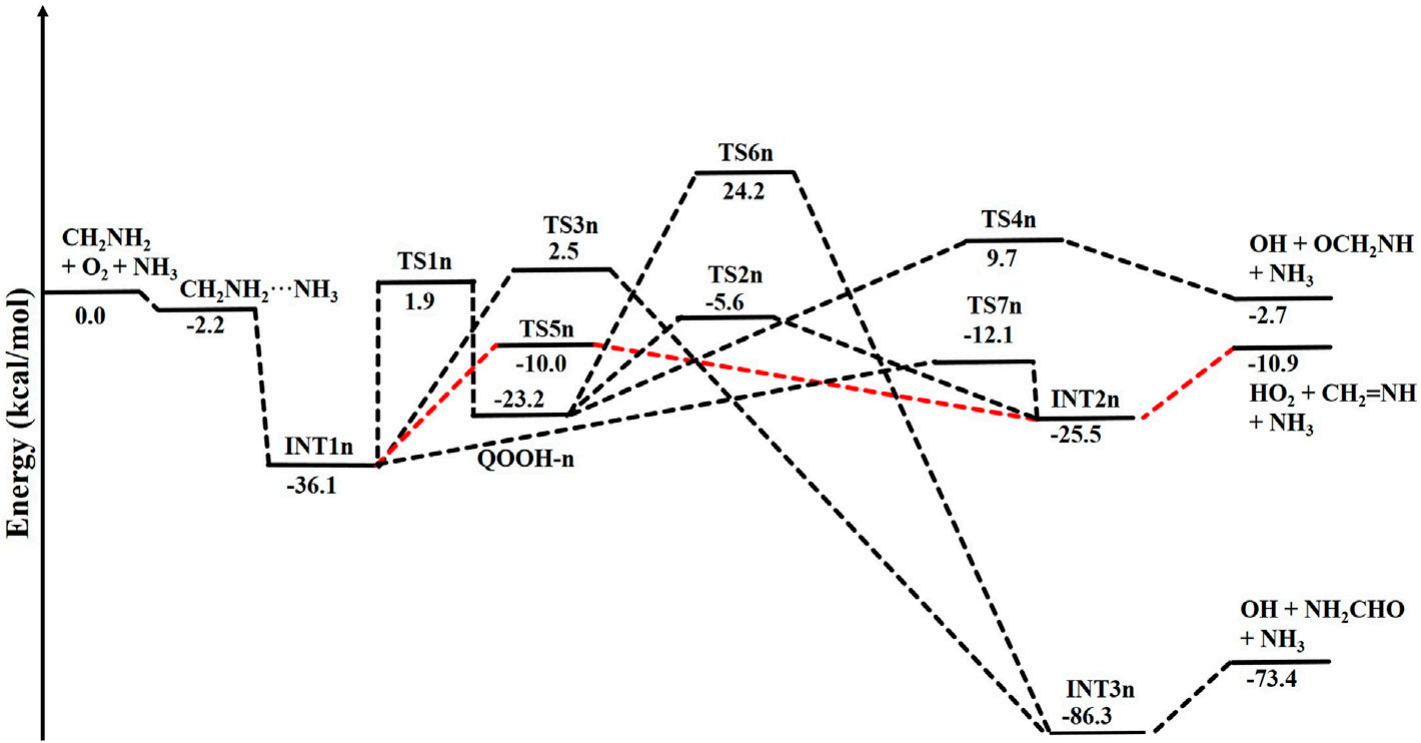
Potential energy surface for the role of ammonia on the ^•^CH_2_NH_2_ + O_2_ reaction obtained using CCSD(T)/6-311++G (3df, 3pd)//M06-2X/6-311++G (3df, 3pd). The energies shown in the figure include the zero-point energy.

When a single ammonia molecule is introduced in ^•^CH_2_NH_2_ + O_2_, the simultaneous collision between ^•^CH_2_NH_2_, O_2_, and NH_3_ is very unlikely to occur; therefore, the probability of a trimolecular reaction is very small under real conditions. Hence, the first step is the formation of a CH_2_NH_2_···NH_3_ complex, followed by collision with O_2_. The CH_2_NH_2_···NH_3_ complex (−2.2 kcal/mol) is assumed to be more important than CH_2_NH_2_···O_2_ and NH_3_···O_2_ due to its lower binding energy (<1 kcal/mol). As discussed in our previous work, we have also used a similar approach for ammonia-assisted reactions (Ali et al., 2019; Ali, 2020; Ali and Balaganesh, 2022; Dash and Ali, 2022). The geometrical changes in ammonia-assisted intermediates and transition states are shown in Figure 4, and the zero-point corrected PES for the ammonia-assisted ^•^CH_2_NH_2_ + O_2_ reaction is given in Figure 5. The energy of all the stationary points, i.e., reactants, intermediates (INTs), and transition states, is tabulated in Table 2.

**TABLE 2:** Enthalpies (in kcal mol^-1^) and entropies (in cal K^−1^ mol^-1^) due to the effect of NH_3_ on each species involved for the ^•^CH_2_NH_2_ + O_2_ reaction.

^•^CH_2_NH_2_ + O_2_ (+NH_3_) →	∆*H*_*rxn*_ (0 *K* )	∆*S*_*rxn*_ (298 *K* )
CH_2_NH_2_···NH_3_	−2.2	−28.2
H_2_NCH_2_OO···NH_3_ (INT1n)	−36.1	−66.3
H···NHCH_2_OO···NH_3_ (TS1n)	1.9	−68.9
HO_2_···CH_2_NH···NH_3_ (TS2n)	−5.6	−70.4
H···CHNH_2_OO···NH_3_ (TS3n)	2.5	−70.9
HO···OCH_2_NH···NH_3_ (TS4n)	9.7	−66.7
H···NHCH_2_···OO···NH_3_ (TS5n)	−10.0	−68.6
H···NHCHO···OH···NH_3_ (TS6n)	24.2	−66.5
H···NHCH_2_···OO···NH_3_ (TS7n)	−12.1	−69.6
HO···OCH_2_NH···NH_3_ (QOOH-n)	−23.2	−68.8
OOH···NHCH_2_···NH_3_ (INT2n)	−25.5	−55.5
HO···NH_2_CHO (INT3n)	−86.3	−61.2

Figure 5 shows that the effect of the ammonia reaction proceeds via similar reaction pathways as a free reaction. For simplicity, only the most stable structures are shown in the PES. As shown in Figure 5, O_2_ attacks the bimolecular complex ^**•**^CH_2_NH_2_···NH_3_ to form a trimolecular hydrogen-bonded complex (INT1n) (Figure 4), whose stabilization energy is −36.1 kcal mol^-1^. The resulting ammonia-assisted intermediate (INT1n) is 4.4 kcal mol^-1^ more stable than the corresponding ammonia-free intermediate, i.e., INT1. This is due to the formation of strong hydrogen bonds between the terminal O-atom of H_2_NCH_2_OO and one of the H-atoms of NH_3_ (2.20 Å) and the H-atom of H_2_NCH_2_OO (2.08 Å) with the N-atom of NH_3_, whereas no such effect is observed in INT1. On the other hand, Table 2 shows that INT1 is entropically more favorable than INT1n with respect to reactants. This is due to the fact that the hydrogen-bonded complex decreases the entropy of the system. Similar to uncatalyzed reaction pathways, the terminal O-atom intra-molecularly attacks the H-atoms in the NH_2_ group in the presence of NH_3_, leading to the formation of INT2n and QOOH-n via five-membered cyclic transition states (TS5n/TS7n and TS1n, respectively). The difference in the barrier heights between two isomeric transition states, TS5n and TS7n, is 2.1 kcal mol^-1^. TS5n seems to be more stable than TS7n because, in the case of TS5n, all three hydrogen atoms of ammonia face toward the molecular center, leading to the formation of a six-membered ring hydrogen-bonded cyclic structure, whereas in the case of TS7n, hydrogen atoms of ammonia are away from the molecular center, leading to the formation of a similar six-membered ring hydrogen-bonded cyclic structure. Entropy data also support that TS5n is more disordered than TS7n.

Supplementary Figure S2 provides an IRC scan at the same level that confirms that TS5n bridges the OCH_2_C(O)OOH radical (INT2n) and CH_2_NH + HO_2_ +NH_3_. The IRC scan confirms that the only stationary point between INT2h and the trimolecular products is that associated. The barrier height of TS1n is 2.5 kcal mol^-1^ lower than that of the corresponding ammonia-free transition state TS1. This is due to the formation of two strong hydrogen bonds (H-atoms of ammonia with N and O atoms of the cyclic ring) in TS1n (1.93 and 2.14 Å). On the other hand, the barrier height of TS5n is almost similar to that of TS5, although hydrogen bonds are present in TS5n. The differences in the barrier height can be explained by the formation of two adjacent cyclic ring structures (five- and six-membered), as aforementioned in TS5n, making it sterically hindered compared to only one ring structure in TS5. The stabilization energies of ammonia-assisted QOOH and INT2 are calculated to be −23.3 and −25.5 kcal mol^-1^, respectively, which are 8.4 and 4.1 kcal mol^-1^ lower than those of the corresponding free-ammonia structures. This can be explained similarly by comparing the presence of hydrogen bonds in the respective structures. In QOOH-n, the formation of two strong hydrogen bonds between the H-atom of ammonia with N atom QOOH (2.26 Å) and N atom NH_3_ and terminal H atom of QOOH (1.76 Å) leads to a seven-membered ring-like structure rather than only one hydrogen bond in the case of free QOOH. Similarly, the stability of INT2n can be explained by the formation of an eight-membered ring with three hydrogen bonds, H of HO_2_ and N of CH_2_NH (1.64 Å), O of HO_2_ and N of NH_3_ (2.1 Å), and H of CH_2_NH and N of NH_3_ (2.24 Å) compared to a six-membered ring with two hydrogen bonds in the case of INT2. On the other hand, the structures of QOOH-n and INT2n are entropically less favorable compared to those of uncatalyzed QOOH and INT2n.

QOOH-n further dissociates to INT2n (via TS2n) and INT3n (via TS6n) and then subsequently forms HO_2_+CH_2_NH + NH_3_ and OH + OCH_2_NH + NH_3_ via TS4n and OH + NH_2_CHO + NH_3_. INT2n and INT3n are eight-membered ring hydrogen-bonded structures, and their stabilization energies are 4.1 kcal mol^-1^ and 7.4 of kcal mol^-1^ lower than those of the corresponding uncatalyzed intermediates. Ammonia-assisted intermediates are more stable than ammonia-free ones because of the formation of an eight-membered ring structure with three strong hydrogen bonds between HO_2_···NH_3_ (2.1 Å), NH_3_···CH_2_NH (2.24 Å), and HO_2_···CH_2_NH (1.64 Å) in INT2n and OH···NH_3_ (2.04 Å), NH_3_···NH_2_CHO (1.87 Å), and HO···NH_2_CHO (1.72 Å) in INT3n. The barrier heights of TS2n (−5.6 kcal mol^-1^), TS3n (2.5 kcal mol^-1^), TS4n (9.7 kcal mol^-1^), and TS6n (24.2 kcal mol^-1^) were also consistently lower than those of the corresponding ammonia-free transition states due to similar hydrogen bonding interactions. Overall, the reaction in the presence of an ammonia-assisted intermediate is thermodynamically more favorable than the free reaction, and *vice versa* entropically.

#### 3.1.3 Role of the water molecule on ^•^CH_2_NH_2_ + O_2_

**FIGURE 6:**
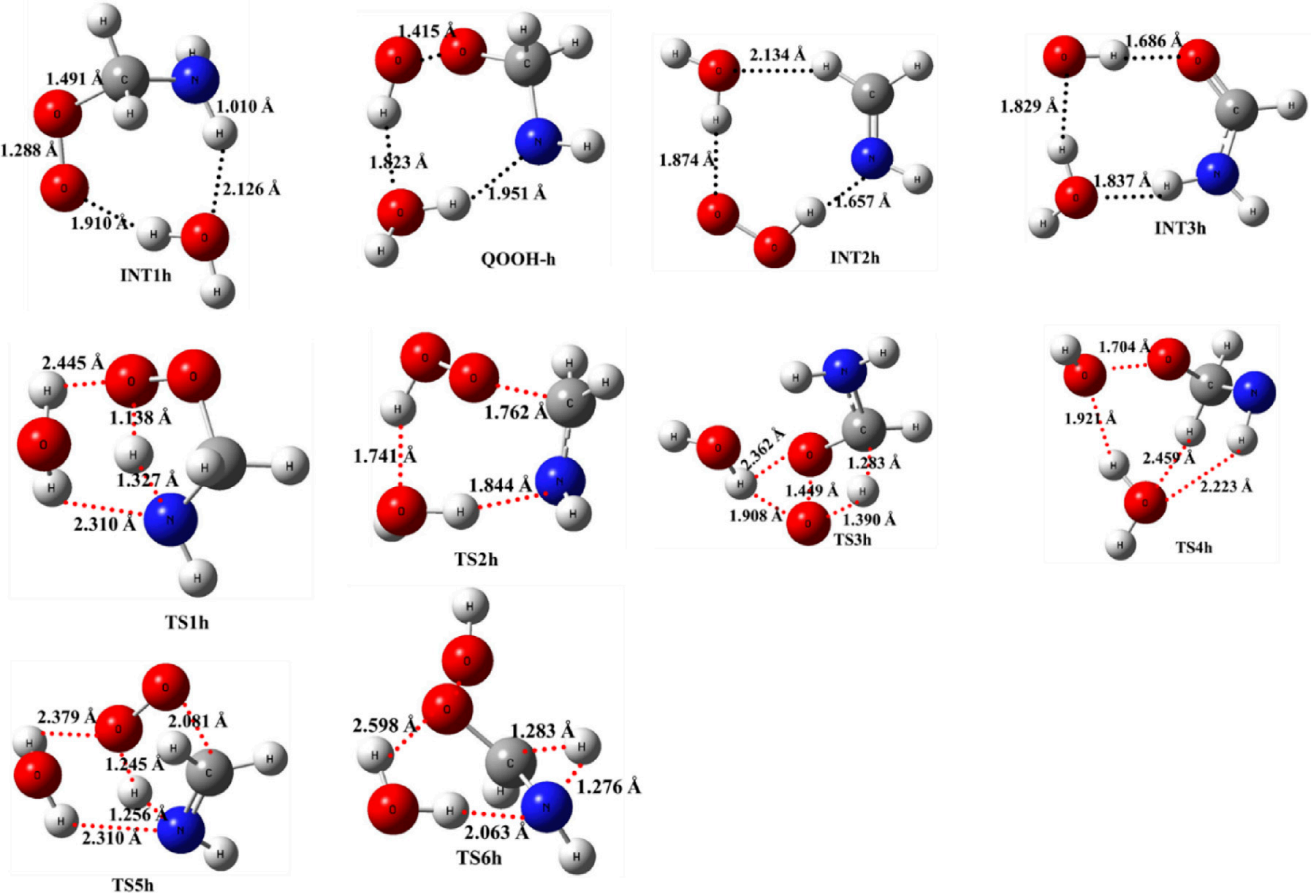
Optimized structures of water-assisted intermediates and transition states were obtained using M06-2X/6-311++G (3df, 3pd).

As previously discussed in the case of ammonia reactions, we have also employed a similar approach for water reactions. When a single H_2_O molecule is added to ^•^CH_2_NH_2_ + O_2_, the first step is the formation of a CH_2_NH_2_···H_2_O complex, followed by collision with O_2_. The CH_2_NH_2_···H_2_O (-2.7 kcal/mol) is assumed to be more important than CH_2_NH_2_···O_2_ and H_2_O···O_2_ (<1 kcal/mol) due to lower binding energy. The geometrical changes in water-assisted intermediates and transition states are shown in Figure 6 and the Cartesian coordinates of all the optimized geometries are given in Supplementary Table S1.

**FIGURE 7:**
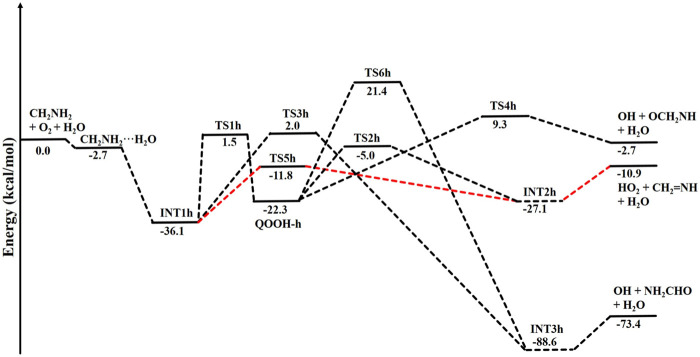
Potential energy surface for the role of water on the ^•^CH_2_NH_2_ + O_2_ reaction obtained using CCSD(T)/6-311++G (3df, 3pd)//M06-2X/6-311++G (3df, 3pd). The energies shown in the figure include the zero-point correction.

The zero-point-corrected PES for the water-assisted ^•^CH_2_NH_2_ + O_2_ reaction is given in Figure 7, and the energy of all the stationary points, i.e*.*, reactants, INTs, and TSs, is tabulated in Table 3. As shown in Figure 6 and Figure 7, the O_2_ molecule attacks the bimolecular complex ^•^CH_2_NH_2_···H_2_O to form a trimolecular hydrogen-bonded complex (INT1h) with a stabilization energy of −36.1 kcal mol^-1^. The INT1h is 4.4 kcal mol^-1^ lower than that of the water-free intermediate (INT1) and identical to the energy of ammonia-assisted INT1n. The result indicates water- and ammonia-assisted reactions are energetically more favorable than free reactions.

**TABLE 3:** Enthalpies (in kcal mol^-1^) and entropies (in cal K^−1^ mol^-1^) due to the effect of H_2_O on each species involved for the ^•^CH_2_NH_2_ + O_2_ reaction.

^•^CH_2_NH_2_ + O_2_ (+H_2_O) →	∆*H*_*rxn*_ (0 *K* )	∆*S*_*rxn*_ (298 *K* )
CH_2_NH_2_···H_2_O	−2.7	−27.9
H_2_NCH_2_OO··· H_2_O (INT1h)	−36.1	−70.3
H···NHCH_2_OO··· H_2_O (TS1h)	1.5	−71.7
HO_2_···CH_2_NH···NH_3_ (TS2h)	−5.0	−71.5
H···CHNH_2_OO···NH_3_ (TS3h)	2.0	−72.0
HO···OCH_2_NH···NH_3_ (TS4h)	9.3	−70.9
H···NHCH_2_···OO···NH_3_ (TS5h)	−11.8	−70.4
H···NHCHO···OH···NH_3_ (TS6h)	21.4	−66.2
HO···OCH_2_NH···NH_3_ (QOOH-h)	−22.3	−71.4
OOH···NHCH_2_···NH_3_ (INT2h)	−27.1	−60.1
HO···NH_2_CHO (INT3h)	−88.6	−64.1

In INT1h, two strong hydrogen bonds are observed between the terminal O-atom of H_2_NCH_2_OO and one of the H-atoms of H_2_O (1.91 Å) and O-atom H_2_O and terminal H-atom H_2_NCH_2_OO (2.12 Å) (see Figure 6). On the other hand, Table 3 and Table 1 show that INT1h is entropically least favored than INT1n and INT1. Similar to the free reaction, the terminal O-atom of INT1h intra-molecularly attacks the H-atoms in the NH_2_ group, leading to the formation of cyclic structures, i.e., INT2h and QOOH-h, via five-membered cyclic transition states, i.e., TS5h and TS1h, respectively. The barrier heights of TS5h and TS1h are 2 kcal mol^-1^ and 3 kcal mol^-1^ lower than those of water-free transition states TS5 and TS1, respectively. In a similar manner, TS5h and TS1h are 1.8 kcal mol^-1^ and 0.4 kcal mol^-1^ lower than TS5n and TS1n, respectively. These differences in barrier heights indicate that water-assisted transition states are energetically more stable than ammonia-assisted and water-free species. The stabilization energies of water-assisted QOOH-h and INT2h are calculated to be −22.3 kcal mol^-1^ and −27.1 kcal mol^-1^, which are 7.8 kcal mol^-1^ and 5.7 kcal mol^-1^ lower than those of the corresponding free structures. This can be understood by comparing the presence of hydrogen bonds in the respective structures. Supplementary Figure S3 provides an IRC scan at the same level that confirms TS5h bridges the OCH_2_C(O)OOH radical (INT2h) and CH_2_NH + H_2_O + HO_2_ products. The IRC scan confirms that the only stationary point between INT2h and the trimolecular products is that associated. In QOOH-h, the formation of two strong hydrogen bonds between the H-atom of water with the N atom of QOOH (1.95 Å) and the O atom of H_2_O and the terminal H atom of QOOH (1.82 Å) led to the formation of a seven-membered ring-like structure. Similarly, the stability of INT2h can be found by the formation of an eight-membered cyclic ring with three hydrogen bonds, H of HO_2_ and N of CH_2_NH (1.65 Å), O of HO_2_ and H of H_2_O (1.87 Å), and H of CH_2_NH and O of H_2_O (2.13 Å), which are compared to that of a six-membered ring with two hydrogen bonds, as indicated in the case of INT2. Between water- and ammonia-catalyzed QOOH and INT2, QOOH-n is more stable than QOOH-h and INT2h is more stable than INT2n.

In general, the water-free pathways are entropically more favorable than water-free ones. The QOOH further dissociates to INT2h (via TS2h) and INT3h (via TS6h) and then subsequently forms HO_2_+ CH_2_NH + H_2_O, OH + OCH_2_NH + H_2_O via TS4h, and OH + NH_2_CHO + H_2_O. In general, the water-assisted reaction channels are thermodynamically more favorable and entropically less favorable than the free reaction. It is also clear from Table 3 that all other pathways are thermodynamically less important compared to R + O_2_→INT1h→TS5h→INT2h→ CH_2_NH + HO_2_+H_2_O, whose barrier height is the lowest with respect to reactants. Therefore, the other reaction channels may have less contribution under tropospheric conditions.

### 3.2 Kinetics

#### 3.2.1 Rate coefficients for the ^•^CH_2_NH_2_ + O_2_ reaction

**FIGURE 8:**
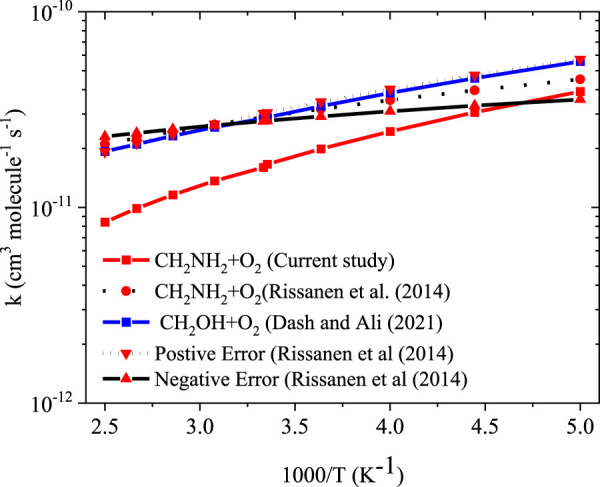
Rate coefficients for the ^•^CH_2_NH_2_ +O_2_ reaction at 1 bar pressure.

To obtain the rate coefficients for ^•^CH_2_NH_2_ + O_2_ in temperatures between 200 K and 400 K and pressures from 0.000001 bar to 1000 bar, the RRKM/ME simulation has been used. The rate coefficients as a function of the temperature at 1 bar pressure are shown in Figure 8. The rate coefficients for the formation of O_2_-CH_2_NH and CH_2_NH are observed to be pressure-dependent and negative temperature-dependent. This result is consistent with that of the previous reports by Rissanen et al. (2014) and a similar reaction system, i.e*.*, ^•^CH_2_OH + O_2_ (Dash and Ali, 2022). The calculated rate coefficient at 300 K (1.6 × 10^−11^ cm^3^ molecule^−1^ s^−1^) is a factor of ∼2 lower than the experimentally measured ones (3.2 × 10^−11^ cm^3^ molecule^−1^ s^−1^) (Rissanen et al., 2014). The calculated value is in good agreement with the lower temperature range <250 K when the positive error is considered. In light of the expected errors in computed thermochemistry, which may be up to 1 kcal/mol, we believe that this level of accuracy is sufficient for these purposes. A recent study by Ali et al. (2023) indicates that the barrier heights of similar reactions are very sensitive to quantum chemical calculations. The computed rate coefficients are compared with previously reported values and also with its isoelectronic analogous reaction system, i.e., ^•^CH_2_OH + O_2_ (see Figure 8) (Dash and Ali, 2022). The calculated values at 300 K are a factor of 2 lower than the theoretically calculated values (Dash and Ali, 2022). The presence of the N atom in CH_2_NH and the O atom ^•^CH_2_OH can explain this, leading to the development of various chemical kinetics conclusions.

**FIGURE 9:**
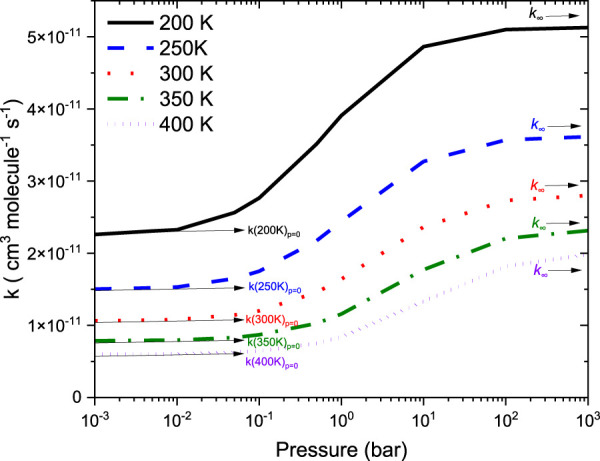
Total rate coefficients as a function in the fall-off regions for the ^•^CH_2_NH_2_ +O_2_ reaction.

**FIGURE 10:**
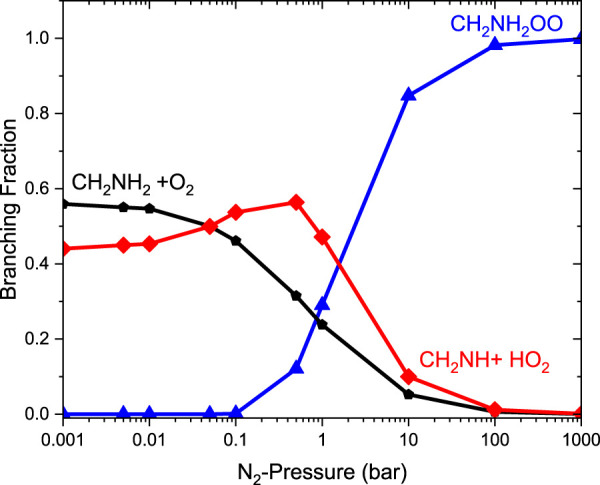
Pressure-dependent branching fractions for the ^•^CH_2_NH + O_2_ reaction at 300 K.

Figure 9 reports the rate coefficients in the fall-off regions for the ^•^CH_2_NH_2_ + O_2_ reaction at different temperatures. The rate coefficients increase as pressure increases, and the HPL condition is observed at ∼100 bar. As shown in Figure 9, the largest difference between the two limits occurs at about 250 K, which reaches a factor of 2. However, at 200 K, the difference between the two regimes is about a factor of 2. To provide more detailed insights, the relative branching fractions of these channels were determined at 200 K, 300 K, and 400 K and shown in Supplementary Figure S4. For simplicity, the branching fraction at different pressures and at 300 K is shown in Figure 10. At all temperatures and pressures, the branching fractions of QOOH are almost negligible; therefore, they are not shown in Figure 10. The branching fraction for the formation CH_2_NH/HO_2_ contributes 40% at 300 K and increases as temperature increases to 400 K. The formation of CH_2_NH decreases to 0% as pressure increases to 100 bar. At the same time, the formation of CH_2_NH_2_OO increases as pressure increases to 100 bar (100%). The plot shows that backward reaction to regenerate reactants is prominent at <0.01 bar. These results are also consistent with the previously reported branching ratio by Rissanen et al. (2014). At all the pressure rates, it is more advantageous to lose HO_2_ via the formation of CH_2_NH rather than through OH loss via formamide formation due to an energetically favorable pathway (see Figure 10). As temperature increases from 300 K to 400 K, the product branching ratio increases (see Supplementary Figure S4). The result is due to the fact that the relevant stationary points on the respective potential energy surfaces have a dominant entropy factor over the enthalpy factor.

#### 3.2.2 Rate coefficients for ^•^CH_2_NH_2_+O_2_ (+NH_3_)

As discussed in the previous section, only the entry channel ^•^CH_2_NH ···NH_3_ + O_2_ is considered for the rate coefficient calculations.

$${\text{CH}}_{2}{\text{NH}}_{2}+O_{2}+{\text{NH}}_{3}\overset{k_{1n}}{\underset{k_{-1n}}{⇌}}{\text{CH}}_{2}{\text{NH}}_{2}---{\text{NH}}_{3}+O_{2}\overset{k_{2n}}{\underset{k_{-2n}}{⇌}}O_{2}-{\text{CH}}_{2}{\text{NH}}_{2}---{\text{NH}}_{3}\overset{k_{\text{uni}-n}}{\to }{\text{CH}}_{2}\text{NH}+{\text{HO}}_{2}+{\text{NH}}_{3}$$

The equation to calculate the effective pressure-dependent rate coefficients $k_{eff}^{bimol}\left(T,M\right)$ is as follows:

$$k_{eff}^{bimol}\left(T,M\right)={K_{eq-1n}\times \left[{NH}_{3}\right]\times K}_{eq-2n}\times k_{\infty }^{uni}\left[1-f_{\left(•CH_{2}NH_{2}\ldots {NH}_{3}+O_{2}\right)}\right],$$ (4)

where $K_{eq-1h}=\frac{k_{1n}}{k_{-1n}}\;and$ and $K_{eq-2n}=\frac{k_{2n}}{k_{-2n}}$ are equilibrium constants of each pathway involved in a reaction, [NH_3_] is the concentration, and *f* is the branching fraction for the reaction proceeding to the reactant. The ammonia concentration used at 10 ppbv is based on the observations from previous studies (Ali et al., 2021). The rate coefficients for the ammonia-assisted reaction are almost similar to those of ^•^CH_2_NH_2_ + O_2_ in the temperature range of 200 K–400 K (see Table 4). In fact, some lower values were obtained at higher temperatures. The catalytic behavior does not take place if step 0 is not included in the reaction mechanism. The result could be due to the lower entropy change in the reaction. The total effective rate coefficient for ^•^CH_2_NH_2_ + O_2_ (2.7 × 10^−21^ cm^3^ molecule^−1^ s^-1^ at 300 K) is ∼12 orders of magnitude lower than that for the ^•^CH_2_NH + O_2_ reaction (8.8 × 10^−13^ cm^3^ molecule^−1^ s^-1^). This result is due to the fact that the ammonia-assisted pathway depends on the ammonia concentration (see Table 4).

**TABLE 4:** Calculated rate coefficients for the ^•^CH_2_NH_2_ + O_2_, ^•^CH_2_NH_2_ +O_2_ (+NH_3_), and ^•^CH_2_NH_2_ +O_2_ (+H_2_O) in the temperature range of 200 K–400 K at 1 bar pressure.

Temperature	^•^CH_2_NH_2_ + O_2_	Exp. value Rissanen et al. (2014)	^32^CH_2_OH + O_2_	^•^CH_2_NH_2_+O_2_ (+NH_3_)	^•^CH_2_NH_2_ +O_2_ (+H_2_O)
200	3.9 × 10^−11^	1.9 × 10^−11^	5.6 × 10^−11^	1.9 × 10^−22^	9.8 × 10^−20^
225	3.1 × 10^−11^	2.2 × 10^−11^	4.6 × 10^−11^	8.6 × 10^−23^	7.8 × 10^−19^
250	2.4 × 10^−11^	2.4 × 10^−11^	3.8 × 10^−11^	4.8 × 10^−23^	3.7 × 10^−18^
275	2.0 × 10^−11^	2.7 × 10^−11^	3.3 × 10^−11^	3.0 × 10^−23^	1.3 × 10^−17^
298	1.7 × 10^−11^	2.9 × 10^−11^	2.8 × 10^−11^	1.9 × 10^−23^	3.3 × 10^−17^
300	1.6 × 10^−11^	2.8 × 10^−11^	2.85 × 10^−11^	1.9 × 10^−23^	3.3 × 10^−17^
325	1.4 × 10^−11^	3.1 × 10^−11^	2.6 × 10^−11^	1.4 × 10^−23^	7.4 × 10^−17^
350	1.2 × 10^−11^	3.4 × 10^−11^	2.3 × 10^−11^	1.1 × 10^−23^	1.4 × 10^−16^
375	0.9 × 10^−11^	3.6 × 10^−11^	2.1 × 10^−11^	8.7 × 10^−24^	2.3 × 10^−16^
400	0.8 × 10^−11^	3.8 × 10^−11^	1.9 × 10^−11^	7.4 × 10^−24^	4.0 × 10^−16^
k = AT^n^	A = 0.02		A = 1.0 × 10^−09^	A = 4.8 × 10^−31^	A = 3.5×10^5^
	n = −3.4		n = −0.75	n = 2.0	n = −6.0
exp (−B/T)	B = 323		B = −222	B = −1858	B = 4958

**FIGURE 11:**
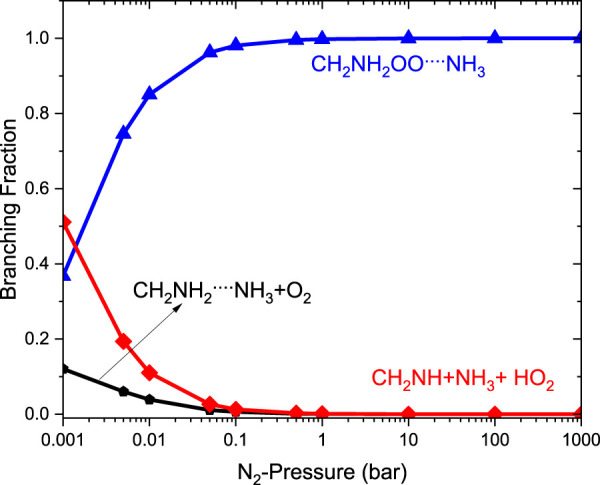
Pressure-dependent branching fractions for the ^•^CH_2_NH_2_ … NH_3_ +O_2_ reaction at 300 K.

The relative branching fractions of these channels determined at 200 K, 300 K, and 400 K are shown in Supplementary Figure S5. For simplicity, the branching fraction at 300 K is shown in Figure 11. The branching fraction for the formation of CH_2_NH/HO_2_ contributes 50% at 300 K, and almost the same temperature increases to 400 K (see Supplementary Figure S5). At the same time, the formation of CH_2_NH_2_OO … NH_3_ increases as the pressure increases to 100 bar (100%). The plot shows the back-reaction that regenerates ^•^CH_2_NH_2_ … NH_3_ + O_2_. When the results are compared with those of the free reaction, it is easier to lose HO_2_ via the formation of CH_2_NH than through OH loss via formamide formation. We can say that the effect of ammonia has a negligible impact on the product branching ratios, and the results are almost similar to those of a free reaction, except at very low pressure (Supplementary Figure S5).

#### 3.2.3 Rate constant for ^•^CH_2_NH_2_ + O_2_ (+H_2_O**)**

The scheme for the formation of INT1h, CH_2_NH, and HO_2_ from ^•^CH_2_NH_2_ + O_2_ reactions with the effect of a water can be written as follows.

$${\text{CH}}_{2}{\text{NH}}_{2}+O_{2}+H_{2}O\overset{k_{1h}}{\underset{k_{-1h}}{⇌}}{\text{CH}}_{2}{\text{NH}}_{2}---H_{2}O+O_{2}\overset{k_{2h}}{\underset{k_{-2h}}{⇌}}O_{2}-{\text{CH}}_{2}{\text{NH}}_{2}–––H_{2}O\overset{k_{\text{uni}-h}}{\to }{\text{CH}}_{2}\text{NH}+{\text{HO}}_{2}+H_{2}O$$

The equation to calculate the effective pressure-dependent rate coefficients $k_{eff}^{bimol}\left(\exp\left(-B/T\right)\right)$ is as follows:

$$k_{eff}^{bimol}\left(T,M\right)={K_{eq-1h}\times \left[H_{2}O\right]\times K}_{eq-2h}\times k_{\infty }^{uni}\left[1-f_{\left(•CH_{2}NH_{2}\ldots H_{2}O+O_{2}\right)}\right],$$ (5)

where $K_{eq\left(1\right)h}=\frac{k_{1h}}{k_{-1h}}$ and $K_{eq-2h}=\frac{k_{2h}}{k_{-2h}}$ are the equilibrium constants of each reaction pathway involved in equation (iii), [H_2_O] is the concentration, and *f* is the branching fraction for the reaction proceeding to the reactants. The [H_2_O] is calculated using a typical humidity concentration, as discussed in the previous paper (Dash and Ali, 2022). The rate coefficients were also calculated using different water concentrations, as shown in Supplementary Figure S6. The effect of relative humidity from 20% to 100% on calculated rate coefficients is a factor of 10 difference. The effective rate coefficient calculated based on Eq. 4 (2.04 × 10^−17^ cm^3^ molecule^−1^ s^-1^ at 298 K) is ∼six to seven orders of magnitude lower than that of the water-free ^•^CH_2_NH_2_ + O_2_ reaction (∼2.2 × 10^−11^ cm^3^ molecule^−1^ s^-1^ at 298 K). This is due to the fact that the water-assisted pathway depends parametrically on water concentration and entropy reduces the rate coefficients. Our results are also consistent with the previously reported values for similar reaction systems (Ali et al., 2022; Dash and Ali, 2022).

**FIGURE 12:**
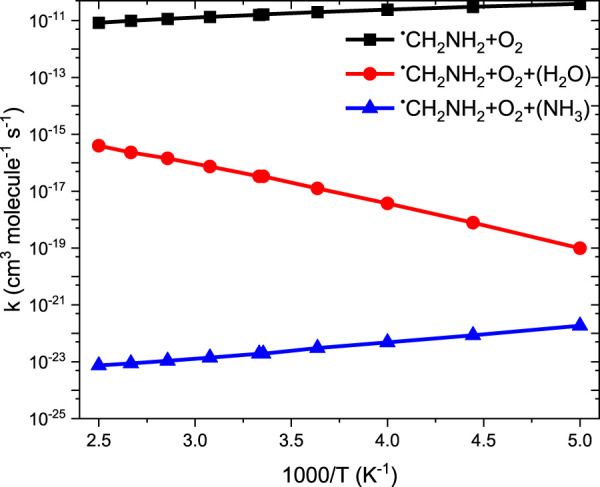
Comparison between rate coefficients for ^•^CH_2_NH_2_ + O_2_, ^•^CH_2_NH_2_ + O_2_ (+NH_3_), and ^•^CH_2_NH_2_ + O_2_ (+H_2_O) in the temperature range of 200 K–400 K at 1 bar pressure.

Our calculation shows that the total effective rate coefficients for systems ^•^CH_2_NH_2_ + O_2_ (+NH_3_) (∼10^–11^ order) and ^•^CH_2_NH_2_ + O_2_ (+H_2_O) (6 order) are smaller than that of the free reaction (see Figure 12).

**FIGURE 13:**
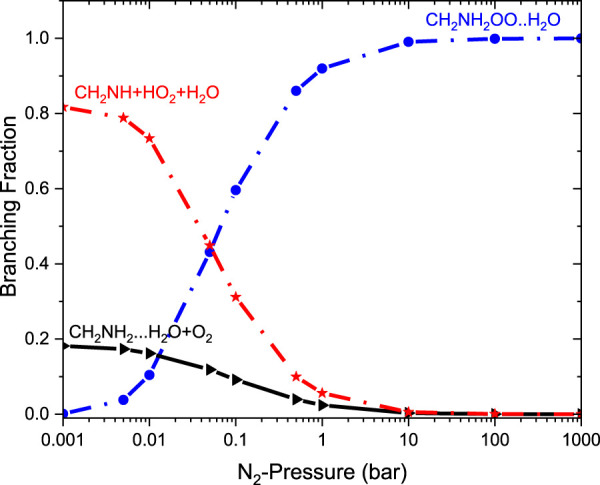
Pressure-dependent branching fractions for the ^•^CH_2_NH_2_ … H_2_O+ O_2_ reaction at 300 K.

It is clear that the geometries of INT and TSs are different in ^•^CH_2_NH_2_ + O_2_ (+NH_3_) reaction systems compared to their isoelectronic analogous ^•^CH_2_NH_2_ + O_2_ (+H_2_O) reactions, resulting in different computed enthalpies and rate coefficients. Because of this, the kinetics of ^•^CH_2_NH_2_ + O_2_ (+NH_3_) is quite different from those of ^•^CH_2_NH_2_ + O_2_ (+H_2_O) reaction systems. In the case of free reactions and ammonia, the rate coefficients exhibit negative temperature dependence, whereas in the case of water, positive temperature dependence was observed. This may be due to the fact that water concentration is highly dependent on temperature, and ammonia concentration is nearly independent of temperature. The branching fractions for the formation of ^•^OOCH_2_NH_2_ and ^•^CH_2_NH and the reaction going back to ^•^CH_2_NH_2_ + O_2_ with the effect of a single water molecule at 300 K and pressure range 0.001 bar–1000 bar are shown in Figure 13, and a comparison of branching fractions for ^•^CH_2_NH_2_ + O_2_, ^•^CH_2_NH_2_ + O_2_ (+NH_3_), and ^•^CH_2_NH_2_ + O_2_ (+H2O) at 200 K, 300 K, and 400 K is shown in Supplementary Figure S7.

As previously discussed in water- and ammonia-free reactions, the product branching ratios for the formation of CH_2_NH + HO_2_ decrease, and the reaction goes back to the reactants, i.e., ^•^CH_2_NH_2_ + O_2_, when the pressure increases from 0.1 bar. When a water molecule is added to the reaction, the product branching ratio changes significantly (∼80%), and a single water reaction favors the formation of CH_2_NH + HO_2_ at a temperature of <300 K; however, the effect of ammonia favors only ∼10%. Despite the slower water reaction, our ME calculations indicate that a favorable CH_2_NH + HO_2_ formation is observed under tropospheric conditions.

### 3.3 Atmospheric fate of methylamine and methanimine

**FIGURE 14:**
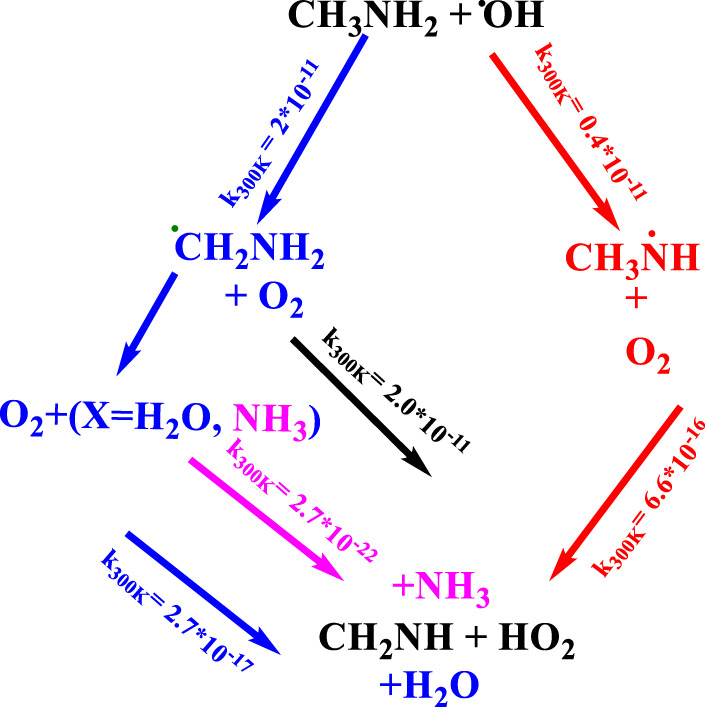
Atmospheric degradation reaction of CH_3_NH_2_ with and without ammonia/water molecules.

The atmospheric degradation of CH_3_NH_2_ with and without ammonia and water molecules is shown in Figure 14. The atmospheric lifetime ( τ ) because of its interaction with OH and species ^•^CH_2_NH_2_ with O_2_ radicals is calculated as follows:

$$\tau =\frac{1}{k_{\left[X=OH,O2\right]}\times \left[X\right]}.$$ (6)

The average OH radical concentration at tropospheric conditions 225 K and <1 bar is ∼1 × 10^6^ cm^3^ molecule^−1^ s^-1,^ and a concentration of [O_2_] = 1 × 10^16^ molecules cm^-3^ was used, which is based on a previous study (Dash and Ali, 2022). We can say that the photo-oxidation lifetime of methylamine to the sink of ^•^CH_2_NH_2_ is nearly 13–14 h. Based on current data and previous results, we can say that the CH_3_NH_2_ + OH/O_2_ → CH_2_O + HO_2_ via ^•^CH_2_NH_2_ leading to the formation of CH_2_NH is both kinetically and thermodynamically more favorable than the CH_3_NH_2_ + OH/O_2_ → CH_3_NH + HO_2_ via CH_3_NH^•^ under tropospheric conditions. This result is also consistent with its isoelectronic analogous (Ali et al., 2019) reaction ^•^CH_2_OH + OH/O_2_ → CH_2_O+ HO_2_. Generally, the effective rate coefficients for the role of water and ammonia reactions are smaller than those of the free ^•^CH_2_NH_2_ + O_2_ reaction system in the temperature range of 200 K–400 K. Therefore, the effect of ^•^CH_2_NH_2_ + O_2_ with H_2_O/NH_3_ is less important for the sink of ^•^CH_2_NH_2_ in a gas-phase atmospheric reaction.

To understand the impact of INT1 in budget calculations, we have calculated the atmospheric lifetime of ∼3 microseconds of ^•^CH_2_NH_2_ with its reaction O_2_, indicating that the formation of CH_2_NH is fast under tropospheric conditions (i.e*.*, at 225 K and an altitude of ∼10–11 km) when taking an average concentration of O_2_ radicals in the upper troposphere of ∼1 × 10^16^ molecule cm^-3^. It is of interest to know whether HO_2_···CH_2_NH_2_···H_2_O can be produced from the reaction of CH_2_NH···H_2_O + O_2_ reaction under atmospheric conditions. For this purpose, we calculated the pseudo-first-order rate coefficients of decay of ^•^CH_2_NH_2_···H_2_O+ O_2_ at 300 K using concentration [O_2_] = 1 × 10^16^ molecules cm^-3^. The decay rate of INT2h producing CH_2_NH_2_+H_2_O-HO_2_ was found to be 8 × 10^−3^ s^-1^, which led to the 2 min of the lifetime of HO_2_···CH_2_NH_2_···H_2_O. Therefore, we can say that under tropospheric conditions, HO_2_···CH_2_NH_2_···H_2_O can be produced from the reaction of ^•^CH_2_NH_2_ + O_2_ (+H_2_O).

To understand the fate of the CH_3_NH^•^ radical with its reaction with O_2_ under the PES (see Supplementary Figure S8), rate coefficients for the addition of O_2_ to CH_3_NH^•^ are investigated using the RRKM/ME simulation. The O_2_ radical mildly reacts with CH_3_NH^•^ with the ∼6 kcal/mol below the reactants. Unlike the formation of common aminoperoxy radicals, O_2_ addition to CH_3_NH^•^ proceeds with a transition state TS1a with ∼5 kcal/mol of barriers, leading to the formation of Int2 and dissected to Int-2 via a five-membered ring transition state with the barrier height of ∼13 kcal/mol (with respect to Int-ad) and dissociated with barrierless process to CH_2_NH + HO_2_. We predicted the rate coefficients using the direct reaction, i.e., CH_3_NH^•^ + O_2_→TS2a→CH_2_NH + HO_2_ (Supplementary Figure S8, in blue), and the indirect reaction, i.e., CH_3_NH^•^ + O_2_ →TS1a→CH_3_NHOO→TS2a→Int2→CH_2_NH + HO_2_ (Supplementary Figure S8 **,** in black). The calculated rate coefficient for the direct formation of CH_2_NH + HO_2_ is 7 × 10^−16^ cm^3^ molecule^−1^ s^-1^, which is at least two orders of magnitude smaller than the indirect reaction. Based on our ME calculation, we can say that the formation of CH_2_NH does not come from the CH_3_NH^•^ +O_2_ reaction because the reaction is quite slow under tropospheric conditions.

The mechanistic and kinetic analysis suggests the formation of these two ^•^CH_2_NH_2_ and CH_3_NH^•^, and our overall understanding of atmospheric and interstellar oxygen chemistry remained uncertain. Although many experimental and computational efforts over the past decade on reaction rate coefficients and branching ratios have been made, our knowledge of the chemical pathways theorized for the O_2_ reaction in two different environments is not clear. Therefore, as suggested in a previous study, at a low temperature, i.e., <100 K, the formation of CH_3_NH^•^ is dominated over the formation of ^•^CH_2_NH_2_ under an interstellar cold medium (Gonza´lez et al., 2022). We have performed the RRKM/ME simulation at below 100 K and found that the formation of CH_3_NOO is dominated under high-pressure limit conditions, i.e., >10 bar and below 100 K. Under low pressure and low temperature, the reaction goes back to the reactant. The formation of CH_3_NOO in the ISM medium is not clear, but our analysis suggests that the reaction CH_3_NH^•^+O_2_ does nothing under ISM conditions. We have also carried out our RRKM/ME simulation under combustion conditions (>1000 K and HPL), and our analysis suggests that the formation of CH_3_NOO^•^ and CH_2_NH is even negligible. It may also be suggested that such a reaction might take place in a more polluted situation.

**FIGURE 15:**
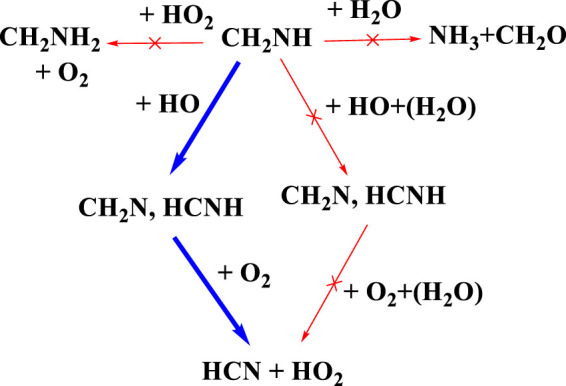
Atmospheric degradation of CH_2_NH with various possible atmospheric species.

There has been considerable speculation about what will happen after the formation of CH_2_NH (Rissanen et al., 2014; Ali et al., 2018; Ali, 2020). Figure 15 shows the atmospheric degradation reaction of CH_2_NH with various possible atmospheric species. The result in the figure shown is based on our previous calculation, except for the reaction of CH_3_NH^•^ + O_2_, which is re-calculated. As shown in Figure 15, we can see that the reaction with water does not lead to the formation of NH_3_ and CH_2_O as suggested in our previous work under tropospheric conditions (Ali, 2020). We also tried to find out if the backward reaction was favorable; for that, we have set up an ME simulation and predicted that this reaction would do nothing in the troposphere (Ali et al., 2016). We also calculated the rate coefficients for the reaction of CH_2_NH + OH radicals, and the mechanism has already been discussed in our previous work (Ali and Barker, 2015). The results show the formation of CH_2_N^•^+ and ^•^CHNH as major products and show a similarity between isoelectronic analogous systems, i.e., CH_2_O and CH_2_CH_2_. As suggested in Rissanen et al. (2014) and Ashraful and Silva (2020), water may favor the formation of CH_2_N^•^ and ^•^CHNH; therefore, we have also analyzed the effect of water molecules on the CH_2_NH + OH reaction (Ali et al., 2019). We have found that the reaction rate coefficients increase when the concentration of water molecules is not included in the calculation and decrease in the presence of water (Ali et al., 2019). Based on our current and previous findings, we propose that the formation of HCN could be the major product when O_2_ radicals react with ^•^CHNH radicals. We also suggest that the gas-phase formation of CH_2_NH from the CH_2_N^•^ + O_2_ reaction will be even slower, as shown in the previous degradation mechanism (see Figure 14). Again, this is due to less favorable N—O-O bond formation than C—O-O bond formation. We can also suggest that the formation of HCN in its presence may be unimportant under tropospheric conditions. Our calculation suggests that oxidation pathways may also contribute to the HO_x_ abundance under the tropospheric conditions, as shown in the degradation reaction mechanism (Figure 15 and Figure 14). Such state-of-the-art kinetics provides a clue to the formation of HCN under tropospheric conditions. Our result may be helpful in setting up an experimental analysis for the formation of HCN under tropospheric conditions.

## 4 Conclusion

In this work, the rate coefficients and branching fraction for ^•^CH_2_NH_2_ + O_2_, ^•^CH_2_NH_2_ + O_2_ (+H_2_O), and ^•^CH_2_NH_2_ + O_2_ (+NH_3_) for the formation of methanimine (CH_2_NH) and HO_2_ have been investigated using CCSD(T)//M06-2X/6-311++G (3df, 3pd) coupled with the RRKM/ME simulation. The results show that ^•^CH_2_NH_2_ + O_2_ leads to the formation of CH_2_NH at temperatures <300 K and goes back to reactants (^•^CH_2_OH + O_2_) at high temperatures (>300 K). When the water/ammonia molecule is added to the ^•^CH_2_NH_2_ + O_2_ reaction, it favors the formation of CH_2_NH at a temperature <300 K. The NH_3_- and H_2_O- assisted rate coefficients are at least 10^10^–10^12^ (Gonza´lez et al., 2022) and 10^6^ times, respectively, smaller than those of the free reaction; thus, we can say that the effect of NH_3_/H_2_O on ^•^CH_2_NH_2_ + O_2_ has less importance in the troposphere. Under tropospheric conditions, the reaction CH_3_NH_2_ + OH/O_2_→ CH_2_NH + HO_2_ via ^•^CH_2_NH_2_ leading to form CH_2_NH + HO_2_ is both kinetically and thermodynamically more favorable than reaction CH_3_NH_2_ + OH/O_2_ → CH_2_NH + HO_2_ via ^•^CH_3_NH. The mechanism indicates that a single NH_3_/H_2_O molecule has the potential to increase the branching fraction in a gas-phase reaction at a lower temperature <300 K and slower reaction at a higher temperature. Such results are promising, and chemical kinetic data can be beneficial for the future implementation of ammonolysis and hydrolysis of other carbon-centered hydroxyl compounds. In previous studies, researchers stated that the reaction CH_2_NH may be favorable in water; our study demonstrated that water increases the formation of CH_2_NH. Our results also indicate the formation of HCN may have come from the reaction going via a carbon-centered radical instead of an N-centered radical. Experimental analysis is required to validate this finding. Such chemical kinetic analysis is interesting; chemical kinetics details can be useful to understand the bigger amine/imine that may lead to the formation of HCN, and N_2_O may increase the reaction rate.

## Data Availability

The original contributions presented in the study are included in the article/Supplementary Material; further inquiries can be directed to the corresponding author.
