# Supplementary material for: Can a single ammonia and water molecule enhance the formation of methanimine under tropospheric conditions?: kinetics of •CH2NH2 + O2 (+NH3/H2O)
Source: Front Chem. 2023 Sep 21;11:1243235. doi: 10.3389/fchem.2023.1243235 (PMC10552757; doi:10.3389/fchem.2023.1243235)
Supplement: Supplementary file 1 [file DataSheet2.docx]

*****Supplementary Materials*****

**Can A Single Ammonia and Water Molecules Enhance the Formation of Methanimine Under Tropospheric Condition?: Kinetics of ^•^CH_2_NH_2_+O_2_ (+NH_3_/H_2_O)**

**Manas Ranjan Dash^3^ and Mohamad Akbar Ali*^1,2^**

^1^Department of Chemistry, College of Art and Science, Khalifa University of Science and Technology, P.O. Box 127788, Abu Dhabi, UAE

^2^Advanced Materials Chemistry Center (AMCC), Khalifa University of Science and Technology, P.O. Box 127788, Abu Dhabi, UAE

^3^Department of Chemistry, School of Physical Sciences, DIT University, Dehradun, Uttarakhand 248009, India

Corresponding author e-mail: [akbar.mohamad@ku.ac.ae](mailto:akbar.mohamad@ku.ac.ae)

*Table of Contents*

Table S1: *Cartesian coordinates of reactants, complexes, products and transition states using M06-2X/6-311++G(3df,3pd) level* …………………………………… *Page 1-16*

**Table S2**: Vibrational parameters of reactants, complexes, products and transition states obtained using M06-2X/6-311++G(3df,3pd) ……………………………………. Page 17-19

**Table S3**: Calculated equilibrium constants (K_eq_ in cm^3^ molecule^-1^) for the formation two body interaction ……….……………………………………………………….Page 20

**Table S4**: Calculated equilibrium constants (K_eq_ in cm^3^ molecule^-1^) for the formation of three-body interaction………………………………………………………………. Page 21

**Figure S1:** Intrinsic reaction coordinate scan connecting to INT1 to INT2 to methanimine + HO_2_ via transition state TS5 calculated at M06-2X/6-311++G(3df,3pd)……………Page 22

**Figure S2:** Intrinsic reaction coordinate scan connecting to INT1n to INT2n to methanimine + HO_2_ +H_2_O *via* transition state TS5_n_ calculated at M06-2X/6-311++G(3df,3pd)……Page 23

**Figure S3:** Intrinsic reaction coordinate scan connecting to INT1h to INT2h to methanimine + HO_2_ +H_2_O *via* transition state TS5h calculated at M06-2X/6-311++G(3df,3pd) ..Page 24

**Figure S4:** Pressure-dependent branching fractions for •CH2NH +O2 reaction………..Page 25

**Figure S5:** Pressure-dependent branching fractions for •CH2NH +O2 + (NH_3_) reaction..Page 26

**Figure S6:** Rate coefficients for CH_2_NH_2_ +O_2_ (+H_2_O) (red color) at different relative humidity (RH) of H_2_O………………………………………………………………………Page 27

**Figure S7:** Comparison of branching fraction for •CH2NH2 +O2 •CH2NH2 +O2 (+NH3) and •CH2NH2 +O2 (+H2O)………………………………………………………………..Page 28

**Figure S8** : PES for CH_3_N+O_2_ reaction……………………………………………….Page 29

Details on the Chemical Kinetics formulation…………………………………………Page 30

•**CH_2_NH_2_**

---------------------------------------------------------------------

Center Atomic Atomic Coordinates (Angstroms)

Number Number Type X Y Z

---------------------------------------------------------------------

1 6 0 0.010445 0.733771 0.000000

2 1 0 0.328182 1.200835 0.925400

3 1 0 0.328182 1.200835 -0.925400

4 7 0 0.010445 -0.663993 0.000000

5 1 0 -0.396073 -1.078172 0.830429

6 1 0 -0.396073 -1.078172 -0.830429

---------------------------------------------------------------------

**O_2_**

---------------------------------------------------------------------

Center Atomic Atomic Coordinates (Angstroms)

Number Number Type X Y Z

---------------------------------------------------------------------

1 8 0 0.000000 0.000000 0.593839

2 8 0 0.000000 0.000000 -0.593839

---------------------------------------------------------------------

**INT1**

---------------------------------------------------------------------

Center Atomic Atomic Coordinates (Angstroms)

Number Number Type X Y Z

---------------------------------------------------------------------

1 6 0 0.568462 0.613604 0.240697

2 1 0 0.437463 0.721324 1.318871

3 1 0 0.938546 1.539106 -0.202534

4 7 0 1.413116 -0.479154 -0.050870

5 1 0 1.025847 -1.335645 0.334443

6 1 0 1.539315 -0.598684 -1.051512

7 8 0 -0.790918 0.498262 -0.317864

8 8 0 -1.364552 -0.579968 0.131944

---------------------------------------------------------------------

**INT2**

---------------------------------------------------------------------

Center Atomic Atomic Coordinates (Angstroms)

Number Number Type X Y Z

---------------------------------------------------------------------

1 7 0 1.377143 -0.102547 0.000000

2 1 0 0.000000 0.989325 0.000000

3 1 0 2.382162 0.081478 0.000000

4 6 0 1.109118 -1.342285 0.000000

5 1 0 0.062357 -1.639719 0.000000

6 1 0 1.871214 -2.127321 0.000000

7 8 0 -1.616930 0.149364 0.000000

8 8 0 -0.959375 1.284108 0.000000

---------------------------------------------------------------------

**INT3**

---------------------------------------------------------------------

Center Atomic Atomic Coordinates (Angstroms)

Number Number Type X Y Z

---------------------------------------------------------------------

1 7 0 0.706664 1.064024 -0.000116

2 1 0 -0.291656 1.260949 -0.000341

3 1 0 1.372892 1.819540 0.000228

4 6 0 1.110234 -0.219585 0.000122

5 1 0 -1.376249 -0.574663 -0.000091

6 1 0 2.205746 -0.340292 0.000444

7 8 0 0.364806 -1.189158 -0.000108

8 8 0 -2.054654 0.152134 0.000088

---------------------------------------------------------------------

**QOOH**

---------------------------------------------------------------------

Center Atomic Atomic Coordinates (Angstroms)

Number Number Type X Y Z

---------------------------------------------------------------------

1 7 0 1.323509 -0.649219 -0.187793

2 1 0 2.266596 -0.627163 0.205395

3 6 0 0.650992 0.534828 0.220711

4 1 0 0.581034 0.572789 1.317284

5 1 0 1.159711 1.439157 -0.133741

6 8 0 -0.630486 0.605835 -0.338164

7 8 0 -1.381533 -0.460796 0.204427

8 1 0 -1.081701 -1.209538 -0.328758

---------------------------------------------------------------------

**TS1**

---------------------------------------------------------------------

Center Atomic Atomic Coordinates (Angstroms)

Number Number Type X Y Z

---------------------------------------------------------------------

1 7 0 -1.074551 -0.688648 -0.057901

2 1 0 0.171359 -1.106631 0.021910

3 1 0 -2.005316 -0.904540 0.263618

4 6 0 -0.696186 0.636911 -0.003928

5 1 0 -0.979298 1.241655 -0.881176

6 1 0 -1.007735 1.184769 0.897481

7 8 0 0.744072 0.672798 0.010373

8 8 0 1.195924 -0.599821 0.005507

---------------------------------------------------------------------

**TS2**

---------------------------------------------------------------------

Center Atomic Atomic Coordinates (Angstroms)

Number Number Type X Y Z

---------------------------------------------------------------------

1 7 0 -1.412248 -0.668443 -0.029007

2 1 0 -1.892659 -0.593449 -0.926129

3 6 0 -0.936744 0.507212 0.319712

4 1 0 -1.244571 1.432256 -0.164308

5 1 0 -0.556382 0.608705 1.329973

6 8 0 0.689455 0.458487 -0.490930

7 8 0 1.520818 -0.290398 0.266938

8 1 0 1.517628 -1.156393 -0.162822

---------------------------------------------------------------------

**TS3**

---------------------------------------------------------------------

Center Atomic Atomic Coordinates (Angstroms)

Number Number Type X Y Z

---------------------------------------------------------------------

1 6 0 -0.374823 -0.005230 0.491114

2 1 0 0.416996 -1.024010 0.442046

3 1 0 -0.505736 0.204937 1.552928

4 1 0 -1.406398 -0.207002 -1.233328

5 8 0 0.625525 0.713622 -0.115188

6 8 0 1.486771 -0.453683 -0.151355

7 7 0 -1.553948 -0.054165 -0.245179

8 1 0 -2.276652 -0.642897 0.140265

---------------------------------------------------------------------

**TS4**

---------------------------------------------------------------------

Center Atomic Atomic Coordinates (Angstroms)

Number Number Type X Y Z

---------------------------------------------------------------------

1 6 0 -0.646383 0.700954 0.000472

2 1 0 -0.787370 1.388673 -0.828842

3 1 0 -0.381221 1.189200 0.936442

4 8 0 0.164385 -0.386838 -0.295532

5 8 0 1.773233 0.075997 0.114379

6 1 0 2.110126 -0.828444 0.153928

7 7 0 -1.571773 -0.387432 0.029049

8 1 0 -1.561763 -0.756408 0.981523

---------------------------------------------------------------------

**TS5**

---------------------------------------------------------------------

Center Atomic Atomic Coordinates (Angstroms)

Number Number Type X Y Z

---------------------------------------------------------------------

1 7 0 1.152029 -0.682443 0.039347

2 1 0 -0.061522 -0.972247 0.206001

3 1 0 1.415166 -0.999074 -0.889285

4 6 0 1.053645 0.617496 0.095272

5 1 0 0.893843 1.084362 1.059333

6 1 0 1.367283 1.263846 -0.717022

7 8 0 -1.019492 0.658362 -0.140762

8 8 0 -1.230614 -0.571457 0.077501

---------------------------------------------------------------------

**TS6**

---------------------------------------------------------------------

Center Atomic Atomic Coordinates (Angstroms)

Number Number Type X Y Z

---------------------------------------------------------------------

1 7 0 1.383945 -0.604753 -0.180938

2 1 0 2.363890 -0.365664 -0.023949

3 6 0 0.637825 0.543094 0.199701

4 1 0 0.919425 -0.421457 0.992489

5 1 0 1.069658 1.537847 0.240111

6 8 0 -0.655828 0.648267 -0.225407

7 8 0 -1.327241 -0.582834 0.003915

8 1 0 -2.002988 -0.299485 0.631649

---------------------------------------------------------------------

**OH**

---------------------------------------------------------------------

Center Atomic Atomic Coordinates (Angstroms)

Number Number Type X Y Z

---------------------------------------------------------------------

1 8 0 0.000000 0.000000 0.107897

2 1 0 0.000000 0.000000 -0.863177

---------------------------------------------------------------------

**OCH_2_NH**

---------------------------------------------------------------------

Center Atomic Atomic Coordinates (Angstroms)

Number Number Type X Y Z

---------------------------------------------------------------------

1 6 0 -0.707472 -0.290127 0.016443

2 1 0 -1.314888 -0.443509 -0.867321

3 1 0 -1.161509 -0.552433 0.966194

4 8 0 0.090767 0.841143 0.023268

5 7 0 0.698964 -0.472402 -0.162077

6 1 0 1.102347 -0.685628 0.750865

---------------------------------------------------------------------

**HO_2_**

---------------------------------------------------------------------

Center Atomic Atomic Coordinates (Angstroms)

Number Number Type X Y Z

---------------------------------------------------------------------

1 1 0 -0.879478 -0.865691 0.000000

2 8 0 0.054967 0.705982 0.000000

3 8 0 0.054967 -0.597770 0.000000

---------------------------------------------------------------------

**CH_2_NH**

---------------------------------------------------------------------

Center Atomic Atomic Coordinates (Angstroms)

Number Number Type X Y Z

---------------------------------------------------------------------

1 1 0 -0.895829 -1.044061 0.000000

2 7 0 0.056304 -0.678692 0.000000

3 6 0 0.056304 0.581482 0.000000

4 1 0 -0.844398 1.197915 0.000000

5 1 0 1.008282 1.108099 0.000000

---------------------------------------------------------------------

**NH_2_CHO**

---------------------------------------------------------------------

Center Atomic Atomic Coordinates (Angstroms)

Number Number Type X Y Z

---------------------------------------------------------------------

1 7 0 1.077820 -0.156418 0.000002

2 1 0 1.174733 -1.158084 -0.000003

3 1 0 1.900374 0.417975 0.000009

4 6 0 -0.163602 0.386856 -0.000011

5 1 0 -0.145542 1.488133 0.000018

6 8 0 -1.186587 -0.246779 0.000004

---------------------------------------------------------------------

**NH_3_**

---------------------------------------------------------------------

Center Atomic Atomic Coordinates (Angstroms)

Number Number Type X Y Z

---------------------------------------------------------------------

1 7 0 0.110617 -0.275363 0.000134

2 1 0 0.489690 -1.214537 -0.000198

3 1 0 0.489681 0.194352 0.813360

4 1 0 0.489722 0.194086 -0.813296

---------------------------------------------------------------------

**CH_2_NH_2_···NH_3_**

---------------------------------------------------------------------

Center Atomic Atomic Coordinates (Angstroms)

Number Number Type X Y Z

---------------------------------------------------------------------

1 6 0 -1.180946 0.688736 0.069264

2 1 0 -0.542349 1.305394 -0.546042

3 1 0 -2.137317 1.080067 0.380527

4 7 0 -1.089648 -0.676721 -0.134862

5 1 0 -0.148358 -1.016651 -0.285324

6 1 0 -1.627831 -1.253686 0.491128

7 7 0 1.864167 -0.039301 0.005112

8 1 0 2.721331 -0.319975 0.464795

9 1 0 1.285812 0.440911 0.687988

10 1 0 2.112749 0.643680 -0.700405

---------------------------------------------------------------------

**INT1n**

---------------------------------------------------------------------

Center Atomic Atomic Coordinates (Angstroms)

Number Number Type X Y Z

---------------------------------------------------------------------

1 6 0 -0.797064 0.604646 0.480988

2 1 0 -0.037596 0.267957 1.184178

3 1 0 -1.641419 1.066058 0.988958

4 7 0 -0.237990 1.442470 -0.488877

5 1 0 0.622667 1.068514 -0.880372

6 1 0 -0.895641 1.745057 -1.191482

7 8 0 -1.409391 -0.637172 -0.056037

8 8 0 -0.515948 -1.496458 -0.412116

9 7 0 2.264695 -0.208975 -0.853986

10 1 0 1.609653 -0.923588 -0.552846

11 1 0 3.007806 -0.152199 -0.169145

12 1 0 2.678182 -0.522955 -1.723129

---------------------------------------------------------------------

**INT2n**

---------------------------------------------------------------------

Center Atomic Atomic Coordinates (Angstroms)

Number Number Type X Y Z

---------------------------------------------------------------------

1 7 0 1.319572 -0.164571 0.051075

2 1 0 -0.037689 0.769385 0.080148

3 1 0 2.246226 0.252256 0.003761

4 6 0 1.350246 -1.428407 0.068080

5 1 0 0.417639 -1.988586 0.113366

6 1 0 2.288704 -1.983697 0.035666

7 8 0 -1.809602 0.392929 -0.271138

8 8 0 -0.916731 1.276455 0.064674

9 7 0 -1.709267 -2.704374 0.004273

10 1 0 -2.101021 -3.201488 -0.786157

11 1 0 -1.843070 -1.711336 -0.166611

12 1 0 -2.270637 -2.945591 0.811951

---------------------------------------------------------------------

**INT3n**

---------------------------------------------------------------------

Center Atomic Atomic Coordinates (Angstroms)

Number Number Type X Y Z

---------------------------------------------------------------------

1 7 0 0.983137 1.257273 -0.030254

2 1 0 0.098487 1.789977 0.020579

3 1 0 1.858390 1.749385 -0.061799

4 6 0 0.995869 -0.076552 -0.066806

5 1 0 -1.660886 -0.429744 0.135910

6 1 0 2.003599 -0.511641 -0.129089

7 8 0 0.012055 -0.796443 -0.037415

8 8 0 -2.612424 -0.173919 0.245131

9 7 0 -1.561171 2.651586 0.103348

10 1 0 -1.752085 3.236951 0.907125

11 1 0 -2.145986 1.820367 0.178088

12 1 0 -1.857543 3.162570 -0.719072

---------------------------------------------------------------------

**QOOH-n**

---------------------------------------------------------------------

Center Atomic Atomic Coordinates (Angstroms)

Number Number Type X Y Z

---------------------------------------------------------------------

1 7 0 1.606616 -0.542613 0.399256

2 1 0 2.426907 -0.124075 0.843073

3 6 0 0.709573 0.523443 0.092537

4 1 0 0.456018 1.080740 1.006104

5 1 0 1.175933 1.216295 -0.621709

6 8 0 -0.455645 0.084402 -0.530688

7 8 0 -1.257599 -0.535320 0.459775

8 1 0 -1.043038 -1.488273 0.290322

9 7 0 -0.230322 -2.912478 -0.365479

10 1 0 -0.265823 -3.864511 -0.027199

11 1 0 0.697906 -2.532210 -0.204868

12 1 0 -0.394200 -2.919932 -1.364151

---------------------------------------------------------------------

**TS1n**

---------------------------------------------------------------------

Center Atomic Atomic Coordinates (Angstroms)

Number Number Type X Y Z

---------------------------------------------------------------------

1 7 0 -1.244427 -0.632762 0.323387

2 1 0 -0.079854 -1.238967 0.123199

3 1 0 -1.989103 -0.585300 1.001327

4 6 0 -0.748766 0.578930 -0.115639

5 1 0 -1.250459 0.982125 -1.012521

6 1 0 -0.687658 1.372577 0.642292

7 8 0 0.609669 0.358907 -0.550516

8 8 0 0.908833 -0.942937 -0.353298

9 7 0 -0.938891 -1.056859 -2.895173

10 1 0 0.001698 -0.755403 -2.671122

11 1 0 -1.402505 -1.246892 -2.014324

12 1 0 -1.416055 -0.286013 -3.345073

---------------------------------------------------------------------

**TS2n**

---------------------------------------------------------------------

Center Atomic Atomic Coordinates (Angstroms)

Number Number Type X Y Z

---------------------------------------------------------------------

1 7 0 -1.541527 -0.548638 0.013595

2 1 0 -2.017748 -0.428318 -0.879945

3 6 0 -0.896985 0.571211 0.314286

4 1 0 -1.140443 1.512796 -0.175961

5 1 0 -0.511528 0.659527 1.324936

6 8 0 0.643738 0.312978 -0.490258

7 8 0 1.476084 -0.332848 0.347252

8 1 0 1.364773 -1.293556 0.061248

9 7 0 0.576825 -2.686830 -0.435235

10 1 0 0.592201 -2.826551 -1.437118

11 1 0 -0.351228 -2.350926 -0.177140

12 1 0 0.749308 -3.575913 0.014681

---------------------------------------------------------------------

**TS3n**

---------------------------------------------------------------------

Center Atomic Atomic Coordinates (Angstroms)

Number Number Type X Y Z

---------------------------------------------------------------------

1 6 0 -0.181886 0.047150 0.333411

2 1 0 0.482165 -0.965723 0.755104

3 1 0 -0.620430 0.478590 1.235313

4 1 0 -0.594639 -0.554053 -1.540853

5 8 0 1.024256 0.611554 -0.029128

6 8 0 1.766294 -0.521989 0.481964

7 7 0 -1.053146 -0.145458 -0.720967

8 1 0 -1.934734 -0.565500 -0.469739

9 7 0 1.128948 -1.012804 -2.422820

10 1 0 1.486621 -1.567092 -3.189730

11 1 0 1.610129 -1.288491 -1.571234

12 1 0 1.399066 -0.048378 -2.579784

---------------------------------------------------------------------

**TS4n**

---------------------------------------------------------------------

Center Atomic Atomic Coordinates (Angstroms)

Number Number Type X Y Z

---------------------------------------------------------------------

1 6 0 -0.655939 0.477998 -0.368637

2 1 0 -1.248079 -0.020353 -1.133210

3 1 0 -0.376384 1.503502 -0.613642

4 8 0 0.422662 -0.272317 0.088876

5 8 0 1.778134 0.193582 -0.855698

6 1 0 2.343068 -0.515890 -0.524110

7 7 0 -1.042257 0.208867 0.978638

8 1 0 -0.623876 0.983234 1.510305

9 7 0 1.090916 2.324546 1.361320

10 1 0 1.554505 1.754982 0.657113

11 1 0 1.572451 2.163659 2.237820

12 1 0 1.237569 3.296339 1.118464

---------------------------------------------------------------------

**TS5n**

---------------------------------------------------------------------

Center Atomic Atomic Coordinates (Angstroms)

Number Number Type X Y Z

---------------------------------------------------------------------

1 7 0 0.903367 -0.822195 -0.610530

2 1 0 0.585032 0.397463 -0.577323

3 1 0 1.913358 -0.863215 -0.711454

4 6 0 0.525290 -1.153982 0.596821

5 1 0 -0.536033 -1.271212 0.783094

6 1 0 1.216056 -1.552722 1.331884

7 8 0 0.325847 0.795937 1.284422

8 8 0 0.389850 1.379996 0.161960

9 7 0 -2.177228 -0.046293 -0.895539

10 1 0 -2.826066 -0.731722 -0.530350

11 1 0 -1.864822 0.531702 -0.125243

12 1 0 -1.371184 -0.538337 -1.262775

---------------------------------------------------------------------

**TS6n**

---------------------------------------------------------------------

Center Atomic Atomic Coordinates (Angstroms)

Number Number Type X Y Z

---------------------------------------------------------------------

1 7 0 1.462856 -0.528332 0.015557

2 1 0 2.423607 -0.187648 0.066949

3 6 0 0.630925 0.598689 0.260910

4 1 0 1.030685 -0.211808 1.172134

5 1 0 0.979428 1.619493 0.142771

6 8 0 -0.682075 0.542114 -0.116185

7 8 0 -1.225540 -0.714928 0.253724

8 1 0 -1.850936 -0.448040 0.938509

9 7 0 -0.374698 -1.450865 -2.527072

10 1 0 0.195632 -1.575336 -1.697021

11 1 0 -1.108742 -0.807934 -2.254412

12 1 0 0.201306 -0.968380 -3.206438

---------------------------------------------------------------------

**TS7n**

---------------------------------------------------------------------

Center Atomic Atomic Coordinates (Angstroms)

Number Number Type X Y Z

---------------------------------------------------------------------

1 7 0 1.001414 -0.528686 -1.056273

2 1 0 -0.162710 -0.923937 -0.831295

3 1 0 0.943881 0.193884 -1.768004

4 6 0 1.272386 -0.026556 0.121735

5 1 0 1.461170 -0.717265 0.934247

6 1 0 1.535383 1.018139 0.251269

7 8 0 -0.725643 -0.010620 0.768376

8 8 0 -1.196634 -0.758686 -0.141457

9 7 0 0.631714 -2.963174 0.821572

10 1 0 0.754852 -3.946558 1.026678

11 1 0 0.862565 -2.822791 -0.156135

12 1 0 -0.352969 -2.748723 0.926505

---------------------------------------------------------------------

**H_2_O**

---------------------------------------------------------------------

Center Atomic Atomic Coordinates (Angstroms)

Number Number Type X Y Z

---------------------------------------------------------------------

1 8 0 1.090345 -0.580966 0.000000

2 1 0 2.048162 -0.544038 0.000000

3 1 0 0.805386 0.334288 0.000000

**---------------------------------------------------------------------**

**CH_2_NH_2_···H_2_O**

---------------------------------------------------------------------

Center Atomic Atomic Coordinates (Angstroms)

Number Number Type X Y Z

---------------------------------------------------------------------

1 6 0 1.104142 0.698330 -0.013816

2 1 0 0.788256 1.254558 0.856451

3 1 0 1.862523 1.118272 -0.656209

4 7 0 1.063919 -0.680345 0.092965

5 1 0 0.248027 -1.053518 0.557624

6 1 0 1.287900 -1.192252 -0.745640

7 8 0 -1.819191 -0.068472 -0.032958

8 1 0 -2.593788 0.490424 0.042596

9 1 0 -1.111678 0.502727 -0.359018

---------------------------------------------------------------------

**INT1h**

---------------------------------------------------------------------

Center Atomic Atomic Coordinates (Angstroms)

Number Number Type X Y Z

---------------------------------------------------------------------

1 6 0 -0.938800 0.630746 0.482475

2 1 0 -0.315504 0.439990 1.353202

3 1 0 -1.928612 0.988881 0.755319

4 7 0 -0.289290 1.489507 -0.408447

5 1 0 0.681291 1.248917 -0.557031

6 1 0 -0.791865 1.652448 -1.267605

7 8 0 -1.261921 -0.711835 -0.080233

8 8 0 -0.206835 -1.422617 -0.282774

9 8 0 2.065996 0.059754 0.534742

10 1 0 1.442826 -0.628584 0.262022

11 1 0 2.936205 -0.339968 0.523557

---------------------------------------------------------------------

**INT2h**

---------------------------------------------------------------------

Center Atomic Atomic Coordinates (Angstroms)

Number Number Type X Y Z

---------------------------------------------------------------------

1 7 0 1.138834 -0.329513 -0.039681

2 1 0 -0.200285 0.643635 0.040683

3 1 0 2.020521 0.132034 -0.254806

4 6 0 1.270968 -1.576733 0.119502

5 1 0 0.395796 -2.176864 0.348778

6 1 0 2.236161 -2.073964 0.030295

7 8 0 -2.051824 0.542889 0.211000

8 8 0 -0.991230 1.280476 0.077070

9 8 0 -1.736161 -2.276804 0.383746

10 1 0 -1.890201 -1.321513 0.313474

11 1 0 -2.582633 -2.657072 0.620454

---------------------------------------------------------------------

**INT3h**

---------------------------------------------------------------------

Center Atomic Atomic Coordinates (Angstroms)

Number Number Type X Y Z

---------------------------------------------------------------------

1 7 0 0.983682 1.232890 -0.128017

2 1 0 0.102343 1.753316 -0.127370

3 1 0 1.848503 1.727815 -0.253797

4 6 0 1.007881 -0.094748 0.002032

5 1 0 -1.599045 -0.383093 0.060401

6 1 0 2.017921 -0.525934 -0.019012

7 8 0 0.030184 -0.811846 0.136377

8 8 0 -2.535154 -0.044971 0.014610

9 8 0 -1.557138 2.538970 -0.196634

10 1 0 -2.080851 1.718483 -0.156857

11 1 0 -1.909310 3.110971 0.486530

---------------------------------------------------------------------

**QOOH-h**

---------------------------------------------------------------------

Center Atomic Atomic Coordinates (Angstroms)

Number Number Type X Y Z

---------------------------------------------------------------------

1 7 0 1.493909 -0.656420 0.167230

2 1 0 2.376802 -0.435114 0.629823

3 6 0 0.693310 0.524447 0.155873

4 1 0 0.521458 0.869747 1.186104

5 1 0 1.200214 1.322991 -0.401927

6 8 0 -0.521507 0.335991 -0.495585

7 8 0 -1.347103 -0.437599 0.355680

8 1 0 -1.217319 -1.330270 -0.020470

9 8 0 -0.275816 -2.671131 -0.821271

10 1 0 -0.207902 -3.510048 -0.363030

11 1 0 0.525596 -2.171947 -0.589627

---------------------------------------------------------------------

**TS1h**

---------------------------------------------------------------------

Center Atomic Atomic Coordinates (Angstroms)

Number Number Type X Y Z

---------------------------------------------------------------------

1 7 0 -1.191667 -0.655265 0.136116

2 1 0 0.012261 -1.151333 -0.119982

3 1 0 -1.942930 -0.763011 0.800032

4 6 0 -0.757839 0.640919 -0.073506

5 1 0 -1.264145 1.156780 -0.907975

6 1 0 -0.759197 1.293438 0.810412

7 8 0 0.619208 0.571362 -0.498602

8 8 0 0.984574 -0.729152 -0.535268

9 8 0 -0.935315 -0.369795 -2.873130

10 1 0 0.017872 -0.365264 -2.752065

11 1 0 -1.251131 -0.929767 -2.157492

---------------------------------------------------------------------

**TS2h**

---------------------------------------------------------------------

Center Atomic Atomic Coordinates (Angstroms)

Number Number Type X Y Z

---------------------------------------------------------------------

1 7 0 -1.441104 -0.641805 0.198420

2 1 0 -1.961701 -0.701881 -0.674975

3 6 0 -0.884418 0.555930 0.310652

4 1 0 -1.222894 1.394403 -0.294811

5 1 0 -0.462047 0.814851 1.276121

6 8 0 0.639947 0.270603 -0.527790

7 8 0 1.544660 -0.184875 0.358573

8 1 0 1.466247 -1.165704 0.275085

9 8 0 0.506290 -2.618111 0.293786

10 1 0 -0.318130 -2.092983 0.389985

11 1 0 0.574967 -3.159757 1.081545

---------------------------------------------------------------------

**TS3h**

---------------------------------------------------------------------

Center Atomic Atomic Coordinates (Angstroms)

Number Number Type X Y Z

---------------------------------------------------------------------

1 6 0 -0.272963 -0.039527 0.425434

2 1 0 0.528674 -1.039719 0.488458

3 1 0 -0.646466 0.084006 1.443316

4 1 0 -0.837197 -0.221530 -1.491154

5 8 0 0.820001 0.746629 0.125423

6 8 0 1.717918 -0.390672 0.174733

7 7 0 -1.234698 -0.071666 -0.564739

8 1 0 -2.036894 -0.647168 -0.357969

9 8 0 0.821198 -0.726148 -2.487764

10 1 0 1.345550 -0.693200 -1.672044

11 1 0 1.156448 -0.007322 -3.026287

---------------------------------------------------------------------

**TS4h**

---------------------------------------------------------------------

Center Atomic Atomic Coordinates (Angstroms)

Number Number Type X Y Z

---------------------------------------------------------------------

1 6 0 -0.678968 0.584034 -0.458579

2 1 0 -1.181280 0.251258 -1.364193

3 1 0 -0.331060 1.615764 -0.484829

4 8 0 0.304844 -0.299130 -0.007703

5 8 0 1.808521 0.277197 -0.565158

6 1 0 2.301971 -0.491449 -0.251275

7 7 0 -1.251109 0.106735 0.758870

8 1 0 -0.883994 0.728824 1.486287

9 8 0 0.971456 1.951489 1.573811

10 1 0 1.482303 1.461519 0.911958

11 1 0 1.454333 2.760703 1.743195

---------------------------------------------------------------------

**TS5h**

---------------------------------------------------------------------

Center Atomic Atomic Coordinates (Angstroms)

Number Number Type X Y Z

---------------------------------------------------------------------

1 7 0 0.788962 -0.750976 -0.641484

2 1 0 0.456184 0.451614 -0.494725

3 1 0 1.791156 -0.767900 -0.805485

4 6 0 0.485224 -1.178200 0.559706

5 1 0 -0.561882 -1.323556 0.795682

6 1 0 1.228445 -1.613220 1.219158

7 8 0 0.290218 0.717738 1.396833

8 8 0 0.258012 1.377592 0.314516

9 8 0 -2.146540 -0.204164 -0.636003

10 1 0 -1.423235 -0.432524 -1.227495

11 1 0 -1.927336 0.671016 -0.307486

---------------------------------------------------------------------

**TS6h**

---------------------------------------------------------------------

Center Atomic Atomic Coordinates (Angstroms)

Number Number Type X Y Z

---------------------------------------------------------------------

1 7 0 1.377451 -0.640133 -0.068800

2 1 0 2.357523 -0.359401 -0.035981

3 6 0 0.610728 0.536851 0.149815

4 1 0 0.964943 -0.272140 1.081458

5 1 0 1.016052 1.532793 0.007896

6 8 0 -0.703064 0.539597 -0.224920

7 8 0 -1.312918 -0.673688 0.191966

8 1 0 -1.961389 -0.341690 0.825089

9 8 0 -0.174003 -1.530187 -2.434108

10 1 0 0.389607 -1.522042 -1.651285

11 1 0 -1.021734 -1.232833 -2.097753

---------------------------------------------------------------------

**Table S2**: Rot-Vibrational parameters of reactants, complexes, products and transition states obtained using M06-2X/6-311++G(3df,3pd). The vibrational frequencies are in cm^-1^ and Rotational constants are in GHZ.

| **CH2NH2** | **Int-1** | **QOOH** | **Int-2** | **TS1** | **TS2** | **TS3** | **TS4** | **TS5** | **TS6** |
| --- | --- | --- | --- | --- | --- | --- | --- | --- | --- |
| 445.76 | 129.78 | 192.77 | 59.28 | -2551.67 | -745.87 | -1548.07 | -1137.81 | -975.21 | -1980.24 |
| 610.42 | 348.73 | 356.21 | 152.38 | 241.65 | 150.45 | 154.32 | 142.52 | 248.89 | 162.48 |
| 667.66 | 421.6 | 408.28 | 196.95 | 339.33 | 263.99 | 351.28 | 161.95 | 362.67 | 176.93 |
| 934.75 | 596.78 | 488.02 | 215.1 | 735.89 | 363.82 | 484.81 | 287.41 | 543.22 | 338.89 |
| 1239.2 | 800.48 | 619.58 | 290.32 | 779.97 | 444.42 | 657.34 | 442.89 | 634.97 | 546.69 |
| 1322.37 | 857.23 | 901.54 | 816.64 | 885.86 | 601.68 | 696.35 | 846.76 | 674.69 | 638.29 |
| 1485.71 | 947.25 | 970.86 | 1093.89 | 930.4 | 977.14 | 956.38 | 958.27 | 1031.11 | 868 |
| 1647.66 | 1191.69 | 1042.01 | 1137.46 | 993.51 | 1053.45 | 997.05 | 1017.03 | 1062.04 | 974.39 |
| 3165.2 | 1247.5 | 1112.52 | 1162.45 | 1050.88 | 1095.3 | 1027.24 | 1043.46 | 1145.49 | 1038.96 |
| 3279.53 | 1309.83 | 1203.8 | 1300.48 | 1101.03 | 1174.39 | 1211.27 | 1161.22 | 1301.66 | 1140.82 |
| 3587.01 | 1387.72 | 1272.47 | 1391.73 | 1175.29 | 1345.63 | 1213.77 | 1244.61 | 1405.16 | 1221.07 |
| 3689.06 | 1413.11 | 1405.03 | 1492.6 | 1226.73 | 1384.68 | 1263.59 | 1317.26 | 1431.42 | 1257.49 |
|  | 1500.06 | 1425.42 | 1644.77 | 1328.92 | 1449.77 | 1421.27 | 1336.42 | 1481.74 | 1390.78 |
|  | 1667.25 | 1439.45 | 1746.21 | 1414.27 | 1559.09 | 1628.05 | 1553.03 | 1640.88 | 1432.47 |
|  | 3113.89 | 2996.68 | 3080.88 | 2076 | 3088.2 | 2002.6 | 3080.81 | 1811.8 | 2291.66 |
|  | 3184.75 | 3052.4 | 3117.79 | 2960.6 | 3194.81 | 3104.1 | 3161.01 | 3133.24 | 3153.91 |
|  | 3561.58 | 3464.04 | 3211.67 | 2995.31 | 3472.86 | 3568.37 | 3458.71 | 3231.6 | 3484.5 |
|  | 3650.2 | 3804.84 | 3512.97 | 3656.64 | 3772.66 | 3678.38 | 3857.64 | 3532.36 | 3834.25 |

|  | **Int-1n** | **QOOH-n** | **Int-2n** | **TS1-n** | **TS2-n** | **TS3-n** | **TS4-n** | **TS5-n** | **TS6-n** |
| --- | --- | --- | --- | --- | --- | --- | --- | --- | --- |
|  | 64.1 | 51.29 | 35.04 | -2576.27 | -762.08 | -1338.52 | -1129.88 | -997.02 | -1972.27 |
|  | 105.41 | 108.03 | 41.69 | 82.35 | 83.46 | 76.32 | 43.08 | 77.32 | 88.71 |
|  | 114.99 | 159.34 | 86.18 | 112.62 | 131 | 144.57 | 129.89 | 107.94 | 115.64 |
|  | 153.94 | 181.5 | 113.8 | 133.63 | 162.4 | 185.66 | 177.81 | 152.78 | 158.07 |
|  | 187.6 | 266.73 | 138.54 | 186.4 | 181.34 | 207.76 | 196.92 | 193.69 | 174.92 |
|  | 210.29 | 291.51 | 167.51 | 227.71 | 310.67 | 240.83 | 216.8 | 198.17 | 197.34 |
|  | 323.54 | 408.98 | 192.56 | 243.39 | 315.99 | 303.79 | 238.36 | 281.13 | 210.62 |
|  | 409.31 | 435.14 | 221.43 | 324.82 | 368.75 | 389.5 | 271.93 | 344.19 | 230.45 |
|  | 481.55 | 490.03 | 242.06 | 358.18 | 405.04 | 493.93 | 312.28 | 390.48 | 326.58 |
|  | 624.74 | 610.34 | 324.01 | 729 | 477.26 | 505.09 | 413.67 | 553.31 | 358.47 |
|  | 798.98 | 895.58 | 362.03 | 770.68 | 617.7 | 675.23 | 449.61 | 640.09 | 557.59 |
|  | 814.95 | 905.99 | 903.85 | 874.44 | 971.37 | 833.26 | 940.69 | 678.11 | 641.07 |
|  | 960.82 | 975.76 | 1090.6 | 910.95 | 1047.23 | 962.22 | 957.84 | 993.69 | 877.07 |
|  | 1068.13 | 1033.96 | 1115.94 | 998.63 | 1068.24 | 1003.03 | 1005.94 | 1028.65 | 973.34 |
|  | 1203.99 | 1081.17 | 1144.81 | 1020.89 | 1119.93 | 1026.48 | 1062.25 | 1070.49 | 1038.85 |
|  | 1253.85 | 1122.86 | 1192.53 | 1044.42 | 1134.88 | 1120.56 | 1103.71 | 1165.92 | 1091.71 |
|  | 1327.4 | 1217.14 | 1327.57 | 1100.28 | 1206.29 | 1209.94 | 1170.81 | 1301.45 | 1137.23 |
|  | 1391.86 | 1280.78 | 1401.09 | 1171.03 | 1329.01 | 1225.34 | 1239.24 | 1402.03 | 1216.57 |
|  | 1405.38 | 1399.17 | 1513.03 | 1219.99 | 1384.35 | 1300.54 | 1326.84 | 1426.53 | 1260.79 |
|  | 1489.93 | 1427.22 | 1650.25 | 1325.32 | 1544.9 | 1423.55 | 1381.89 | 1471.7 | 1385.9 |
|  | 1642.01 | 1606.14 | 1669.33 | 1387.2 | 1626.7 | 1630.39 | 1542.92 | 1628.71 | 1429.14 |
|  | 1665.9 | 1641.44 | 1677.26 | 1646.15 | 1651.9 | 1636.56 | 1649.48 | 1652.24 | 1655.97 |
|  | 1668.53 | 1660.48 | 1744.77 | 1665.41 | 1675.74 | 1659.17 | 1669.9 | 1671.24 | 1678.89 |
|  | 3088.8 | 2977.69 | 2905.01 | 2060.92 | 2974.3 | 1993.92 | 3072.47 | 1802.81 | 2289.44 |
|  | 3164.37 | 3028.92 | 3058.12 | 2937.79 | 3073.58 | 3085.61 | 3157.68 | 3130.79 | 3158.17 |
|  | 3460.15 | 3254.51 | 3175.78 | 3002.85 | 3181.83 | 3320.41 | 3382.32 | 3235.32 | 3481.25 |
|  | 3485.82 | 3462.32 | 3475.89 | 3501.35 | 3427.5 | 3478.82 | 3464.4 | 3499.75 | 3491.66 |
|  | 3609.97 | 3486.3 | 3503.78 | 3627.51 | 3478.76 | 3590.66 | 3590.72 | 3530.33 | 3604.88 |
|  | 3633.07 | 3614.1 | 3597.61 | 3641.28 | 3589.98 | 3634.41 | 3630.22 | 3629.93 | 3628.55 |
|  | 3638.56 | 3651.09 | 3630.07 | 3648.55 | 3650.04 | 3647.96 | 3843.98 | 3645.39 | 3826.22 |
| **1D=A** | 5.40785 | 5.7015 | 3.09816 | 5.55275 | 5.37403 | 5.05183 | 4.43785 | 4.7479 | 5.13816 |
| **B** | 3.18421 | 3.46159 | 3.00549 | 3.40521 | 3.51844 | 4.0547 | 4.05316 | 3.37336 | 3.36795 |
| **C** | 2.19076 | 2.45353 | 1.54566 | 2.82827 | 2.35213 | 2.56642 | 2.44141 | 2.52695 | 2.46988 |
| **2D= (B*C)^0.5^** | 2.641182 | 2.914295 | 2.155334 | 3.103362 | 2.876774 | 3.225843 | 3.1457 | 2.919642 | 2.884169 |

|  | **Int-1h** | **QOOHh** | **Int-2h** | **TS1h** | **TS2h** | **TS3h** | **TS4h** | **TS5h** | **TS6h** | **CH2NH2--H2O** |
| --- | --- | --- | --- | --- | --- | --- | --- | --- | --- | --- |
|  | 108.03 | 104.86 | 56.53 | -2539.44 | -735.47 | -1295.85 | -1127.5 | -965.75 | -1972.79 | 112.21 |
|  | 173.08 | 163.65 | 78.88 | 119.98 | 103.37 | 111.19 | 158.38 | 88.92 | 74.64 | 155.61 |
|  | 187.91 | 202.29 | 94.59 | 148.83 | 153.35 | 185.25 | 187.99 | 158.82 | 130.17 | 181.73 |
|  | 202.97 | 252.52 | 161.21 | 199.23 | 210.57 | 204.18 | 212.77 | 194.12 | 158.29 | 194.49 |
|  | 230.64 | 300.8 | 192.41 | 250.82 | 260.41 | 242.47 | 222.04 | 254.99 | 179.62 | 320.29 |
|  | 383.15 | 370.63 | 211.96 | 273.31 | 315.43 | 275.11 | 259.16 | 293.67 | 205.75 | 474.46 |
|  | 407.18 | 436.42 | 228.77 | 349.89 | 324.14 | 356.69 | 287.47 | 335.95 | 216.59 | 518.83 |
|  | 448.92 | 489.65 | 256.61 | 392.88 | 395.18 | 461.76 | 328.81 | 383.34 | 308.64 | 682.05 |
|  | 551.59 | 554.69 | 301.74 | 477.53 | 469.67 | 502.13 | 400.72 | 438.89 | 362.2 | 737.2 |
|  | 633.47 | 610.2 | 428.31 | 740.35 | 634.84 | 681.07 | 462.93 | 550.72 | 487.08 | 949.91 |
|  | 740.12 | 825.19 | 574.15 | 774.46 | 694.37 | 719.61 | 654.11 | 654.77 | 571.71 | 1249.75 |
|  | 800.46 | 903.54 | 910.07 | 881.41 | 928.43 | 804.81 | 905.56 | 676.1 | 646.87 | 1329.34 |
|  | 953.71 | 973.17 | 1116.79 | 914.44 | 1014.02 | 960.07 | 948.79 | 1027.54 | 882.91 | 1479.74 |
|  | 1206.66 | 1028.83 | 1144.45 | 1011.66 | 1063.25 | 1001.59 | 1021.66 | 1064.19 | 977.32 | 1622.3 |
|  | 1251.78 | 1121.58 | 1174.25 | 1064.6 | 1119.54 | 1023.83 | 1061.6 | 1174.21 | 1038.95 | 1647.16 |
|  | 1327.32 | 1214.12 | 1335.53 | 1106.92 | 1203.82 | 1203.6 | 1169.71 | 1302.64 | 1143.21 | 3147.13 |
|  | 1382.19 | 1280.95 | 1408.03 | 1183.79 | 1334.69 | 1217.62 | 1233.5 | 1397.17 | 1220.52 | 3266.47 |
|  | 1409.57 | 1398.92 | 1514.67 | 1220.68 | 1382.62 | 1298.19 | 1329.05 | 1428.03 | 1259.95 | 3561.19 |
|  | 1492.74 | 1425.33 | 1654.28 | 1328.85 | 1540.92 | 1420.1 | 1361.53 | 1472.15 | 1385.99 | 3674.75 |
|  | 1634.07 | 1558.43 | 1677.06 | 1389.74 | 1596.96 | 1634.39 | 1545.4 | 1622.53 | 1424.14 | 3779.33 |
|  | 1650.61 | 1637.8 | 1749.78 | 1647.36 | 1639.05 | 1649.19 | 1633.03 | 1647.5 | 1656.91 | 3960.83 |
|  | 3095.93 | 2985.17 | 2889.95 | 2060.37 | 3086.72 | 1987.18 | 3078.57 | 1799.89 | 2285.86 |  |
|  | 3176.53 | 3039.61 | 3093.99 | 2938.43 | 3187.55 | 3089.96 | 3166.81 | 3127.49 | 3184.03 |  |
|  | 3555.16 | 3471.25 | 3222.41 | 3006.58 | 3318.4 | 3451.64 | 3448.95 | 3237.61 | 3499.08 |  |
|  | 3668.59 | 3575.32 | 3494.33 | 3651.33 | 3453.92 | 3649.5 | 3721.61 | 3529 | 3819.34 |  |
|  | 3740.6 | 3681.95 | 3696.33 | 3863.92 | 3494.71 | 3715.71 | 3843.09 | 3862.13 | 3845.27 |  |
|  | 3969.58 | 3956.64 | 3967.92 | 3948.52 | 3945.26 | 3955.9 | 3972.03 | 3961.98 | 3949.73 |  |
| **1D=A** | 5.32418 | 5.60106 | 3.81707 | 5.58217 | 5.17927 | 5.16444 | 4.90832 | 4.80812 | 5.10139 | 24.55764 |
| **B** | 3.67222 | 3.69814 | 2.84089 | 3.69602 | 3.89207 | 4.1146 | 4.0992 | 3.59645 | 3.65555 | 5.07416 |
| **C** | 2.4431 | 2.49409 | 1.62973 | 3.01428 | 2.44058 | 2.59461 | 2.57055 | 2.6495 | 2.5695 | 4.37195 |
| **2D= (B*C)^0.5^** | 2.995263 | 3.037021 | 2.151716 | 3.33779 | 3.08203 | 3.267382 | 3.246105 | 3.086875 | 3.06479 | 4.7099866 |

**Table S3**: Calculated equilibrium constants (K_eq_ in cm^3^ molecule^-1^) for two body interaction

(a) ^•^CH_2_NH_2_ +O_2_🡺CH_2_NH_2_OO

| Temp | Ke |
| --- | --- |
| 200 | 2.24E+08 |
| 225 | 2.63E+04 |
| 250 | 1.87E+01 |
| 275 | 4.92E-02 |
| 298.15 | 4.88E-04 |
| 300 | 3.48E-04 |
| 325 | 5.26E-06 |
| 350 | 1.45E-07 |
| 375 | 6.42E-09 |
| 400 | 4.21E-10 |

**Table S3 (b)**: Calculated equilibrium constants (K_eq_ in cm^3^ molecule^-1^) for ^•^CH_2_NH_2_ +NH_3_ 🡺CH_2_NH--NH_3_

| Temp | Ke |
| --- | --- |
| 200 | 3.29E-23 |
| 225 | 1.75E-23 |
| 250 | 1.08E-23 |
| 275 | 7.39E-24 |
| 298.15 | 5.60E-24 |
| 300 | 5.49E-24 |
| 325 | 4.33E-24 |
| 350 | 3.58E-24 |
| 375 | 3.07E-24 |
| 400 | 2.72E-24 |

**Table S3 (C)**: Calculated equilibrium constants (K_eq_ in cm^3^ molecule^-1^) for ^•^CH_2_NH_2_ +H_2_O 🡺CH_2_NH--H_2_O

| Temp | Ke |
| --- | --- |
| 200 | 1.34E-22 |
| 225 | 5.994E-23 |
| 250 | 3.206E-23 |
| 275 | 1.953E-23 |
| 298.15 | 1.346E-23 |
| 300 | 1.311E-23 |
| 325 | 9.479E-24 |
| 350 | 7.265E-24 |
| 375 | 5.832E-24 |
| 400 | 4.858E-24 |

**Table S4**: Calculated equilibrium constants (K_eq_ in cm^3^ molecule^-1^) for three body interaction

(a) ^•^CH_2_NH_2_ ^….^NH_3_+ O_2_ 🡺 INTn

| Temp | Ke-Int-1n |
| --- | --- |
| 200 | 2.52E+10 |
| 225 | 1.67E+06 |
| 250 | 7.52E+02 |
| 275 | 1.37E+00 |
| 298.15 | 1.02E-02 |
| 300 | 7.11E-03 |
| 325 | 8.29E-05 |
| 350 | 1.83E-06 |
| 375 | 6.69E-08 |
| 400 | 3.71E-09 |

(b) ^•^CH_2_NH^…^H_2_O+ O_2_ 🡺 INTh

| Temp | Ke-Int-1n |
| --- | --- |
| 200 | 2.00E+09 |
| 225 | 1.40E+05 |
| 250 | 6.54E+01 |
| 275 | 1.22E-01 |
| 298.15 | 9.30E-04 |
| 300 | 6.50E-04 |
| 325 | 7.71E-06 |
| 350 | 1.72E-07 |
| 375 | 6.38E-09 |
| 400 | 3.57E-10 |

**Figure S1:** Intrinsic reaction coordinate scan connecting to INT1 to INT2 to methanimine + HO_2_ via transition state TS5 calculated at M06-2X/6-311++G(3df,3pd).

**Figure S2:** Intrinsic reaction coordinate scan connecting to INT1n to INT2n to methanimine + HO_2_ +H_2_O *via* transition state TS5n calculated at M06-2X/6-311++G(3df,3pd).

**Figure S3:** Intrinsic reaction coordinate scan connecting to INT1h to INT2h to methanimine + HO_2_ +H_2_O *via* transition state TS5h calculated at M06-2X/6-311++G(3df,3pd).

**Figure S4:** Pressure-dependent branching fractions for •CH2NH +O2 reaction at 200 (dashed line), 300 K (solid line) and 400K (dotted lines).

**Figure S5:** Pressure-dependent branching fractions for •CH2NH2…NH3 +O2 reaction at 200 (dashed line), 300 K (solid line) and 400K (dotted lines).

**Figure S6:** Rate coefficients for CH_2_NH_2_ +O_2_ (+H_2_O) (red color) at different relative humidity (RH) of H_2_O

**Figure S7**: Comparison of branching fraction for free •CH2NH2 +O2 (black color), •CH2NH2 +O2 (+NH3) blue color) and •CH2NH2 +O2 (+H2O) (red color) at 298 K. The temperature 200 K (dashed line), 300 K (solid line) and 400K (dotted lines)


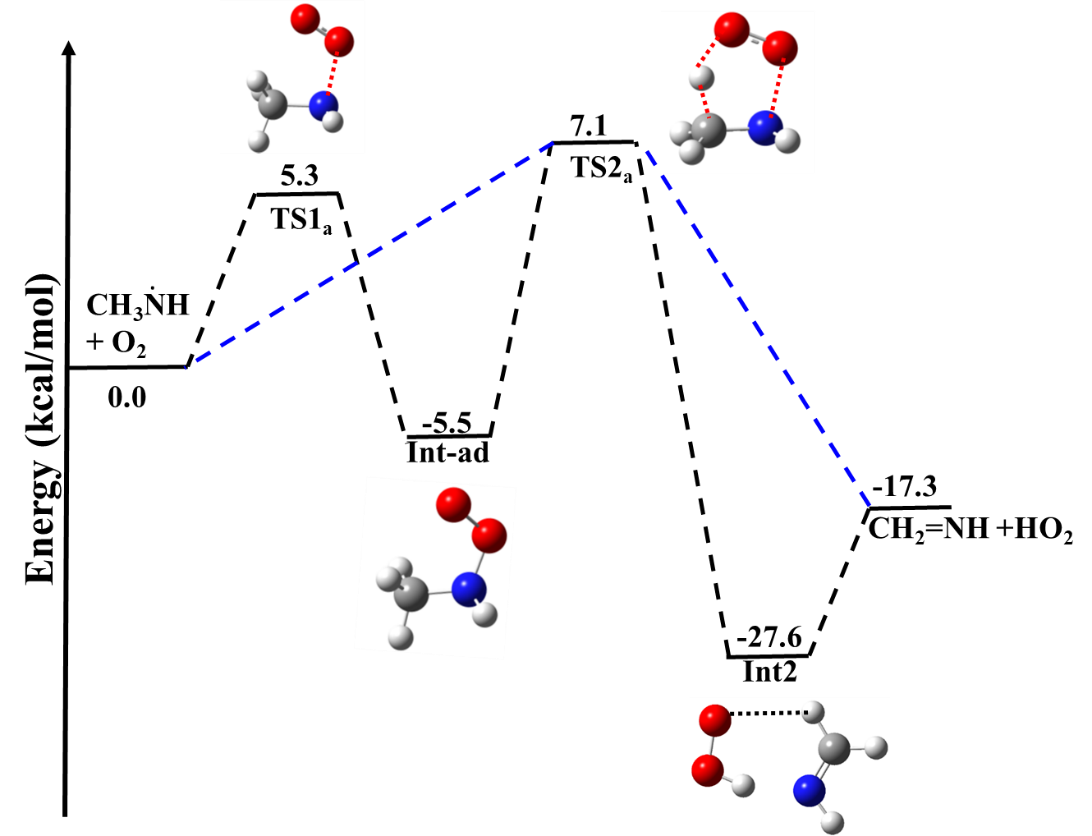


**Figure S8**: PES for CH_3_NH+O_2_ reaction. The energies shown in the figure include zero-point correction.

**Chemical Kinetics Calculations**

All the kinetics calculations were done using software tool in the Multiwell Suite of the program.^49-51^ The “me” codes calculate the unimolecular rate coefficients k *(E*) based on Rice−Ramsperger−Kassel−Marcus (RRKM)/master equation (ME) is given in eqn (1):^52^

$k\left( E \right)=[\frac{m^{\neq}}{m}\frac{\sigma_{ext}}{\sigma_{ext}^{\neq}}]\frac{g_{e}^{\neq}}{g_{e}}\frac{1}{h}\frac{G^{\neq}(E-E_{0,0})}{\rho(E)}$ (1)

To avoid the reppition from the previous studies the details of each terms are given in the supporting information. where $m^{\neq}$ and m are the number of optical isomers, $\sigma_{ext}^{\neq}$ and $\sigma_{ext}$ are the external rotation symmetry numbers, and $g_{e}^{\neq}$and $g_{e}$ are the electronic state degeneracies of the transition state and reactant, respectively. The quantity set off square brackets is the reaction path degeneracy, *h* is Planck’s constant; $\rho(E,J)$is the density of states of the reactant molecule; $G^{\neq}(E-E_{0,J},J)$ is the sum-of-states of the transition state; ${\mathrm{and}E}_{0,J}$is the reaction critical energy, which depends on angular momentum. The obtained vibrational frequencies and the rotational constants from M06-2X/6-311++G(3df,3pd) were applied to compute the density of states and the sum of states based on the Stein–Rabinovitch version of the Beyer–Swinehart algorithm.^58^

$G^{\neq}\left( E-E_{0,0} \right)=\sum_{J=0}^{Jmax} G^{\neq}(E-E_{0,J},J)$ (2)

$\rho(E,J)=\sum_{J=0}^{Jmax} \rho_{i}(E,J)$ (3)

where$E_{0,0}$ is the critical energy at angular momentum *J=0*. This energy includes zero-point correction and centrifugal corrections at temperature T. In this work, the molecular rotation of each species involved in the reaction were treated as the symmetric tops approximation for the rotational constants A>B=C) with a 2-D rotor or J-rotor with rotational constant was calculated by (BC)^1/2^. 2D-rotor (B=C) was assumed to be adiabatic, and a one-dimensional (1D) rotor (the K-rotor with rotational constant A) is assumed to be an active degree of freedom.

The pressure-dependent product branching ratios were simulated using estimated energy transfer parameters for the intermediates.

58. Stein, S. E., and Rabinovitch, B. S., (1973). Accurate evaluation of internal energy level sums and densities including anharmonic oscillators and hindered rotors, *J. Chem. Phys.* 58, 2438–2445. doi.org/10.1063/1.1679522
