# Supplementary figures and images for: Can a single ammonia and water molecule enhance the formation of methanimine under tropospheric conditions?: kinetics of •CH2NH2 + O2 (+NH3/H2O)
Source: Front Chem. 2023 Sep 21;11:1243235. doi: 10.3389/fchem.2023.1243235 (PMC10552757; doi:10.3389/fchem.2023.1243235)

**Atmospheric Production of Hydrogen cyanide**

**
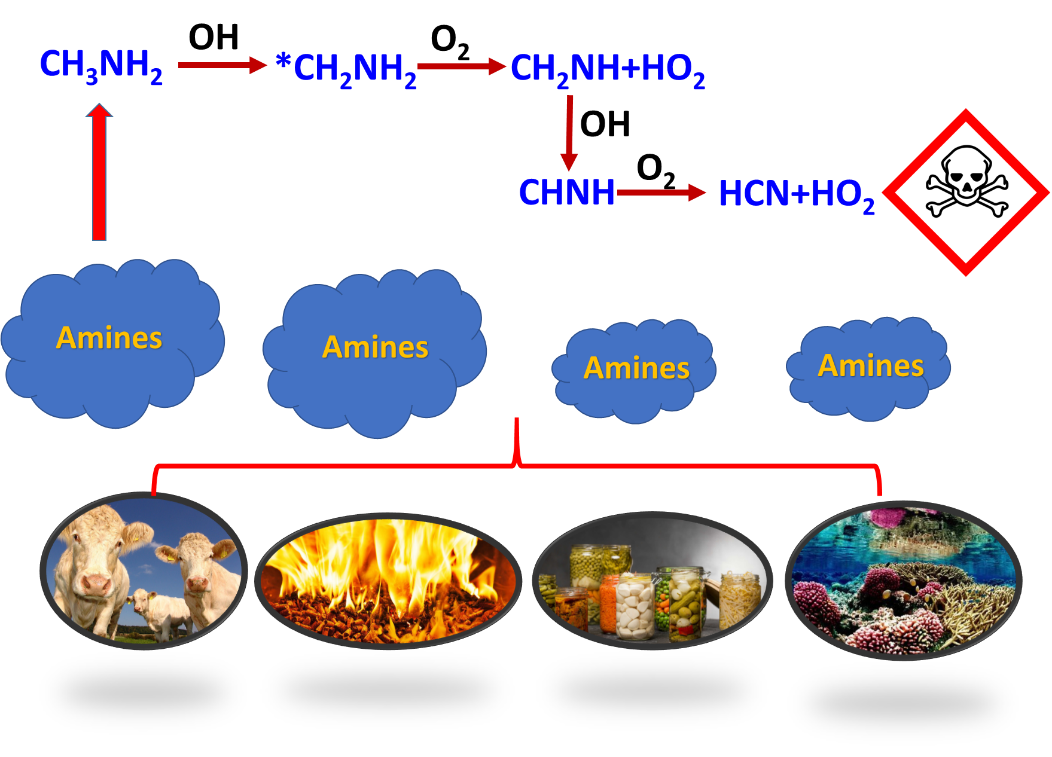
**

Supplement: Supplementary file 2 [file DataSheet1.docx]
